# LncRNA SNHG8 is identified as a key regulator of acute myocardial infarction by RNA-seq analysis

**DOI:** 10.1186/s12944-019-1142-0

**Published:** 2019-11-18

**Authors:** Liu-An Zhuo, Yi-Tao Wen, Yong Wang, Zhi-Fang Liang, Gang Wu, Mei-Dan Nong, Liu Miao

**Affiliations:** Department of Cardiology, Institute of Cardiovascular Diseases, the Liu Zhou People’s Hospital, 8 Wenchang Road, Liuzhou, 545006 Guangxi China

**Keywords:** Acute myocardial infarction, LncRNA-miRNA-mRNA regulators, Triple network analyses, Functional enrichment, Diagnostic biomarker

## Abstract

**Background:**

Long noncoding RNAs (lncRNAs) are involved in numerous physiological functions. However, their mechanisms in acute myocardial infarction (AMI) are not well understood.

**Methods:**

We performed an RNA-seq analysis to explore the molecular mechanism of AMI by constructing a lncRNA-miRNA-mRNA axis based on the ceRNA hypothesis. The target microRNA data were used to design a global AMI triple network. Thereafter, a functional enrichment analysis and clustering topological analyses were conducted by using the triple network. The expression of lncRNA SNHG8, SOCS3 and ICAM1 was measured by qRT-PCR. The prognostic values of lncRNA SNHG8, SOCS3 and ICAM1 were evaluated using a receiver operating characteristic (ROC) curve.

**Results:**

An AMI lncRNA-miRNA-mRNA network was constructed that included two mRNAs, one miRNA and one lncRNA. After RT-PCR validation of lncRNA SNHG8, SOCS3 and ICAM1 between the AMI and normal samples, only lncRNA SNHG8 had significant diagnostic value for further analysis. The ROC curve showed that SNHG8 presented an AUC of 0.850, while the AUC of SOCS3 was 0.633 and that of ICAM1 was 0.594. After a pairwise comparison, we found that SNHG8 was statistically significant (*P*_SNHG8-ICAM1_ = 0.002; *P*_SNHG8-SOCS3_ = 0.031**)**. The results of a functional enrichment analysis of the interacting genes and microRNAs showed that the shared lncRNA SNHG8 may be a new factor in AMI.

**Conclusions:**

Our investigation of the lncRNA-miRNA-mRNA regulatory networks in AMI revealed a novel lncRNA, lncRNA SNHG8, as a risk factor for AMI and expanded our understanding of the mechanisms involved in the pathogenesis of AMI.

## Background

Acute myocardial infarction (AMI), one of the leading causes of mortality and morbidity worldwide, is a manifestation of acute coronary syndrome (ACS) [1]. ACS is a group of clinical syndromes that include unstable angina pectoris, non-ST-segment elevation AMI (NSTEMI), ST-segment elevation AMI (STEMI) and sudden death [2]. Many associated risk factors, such as age, sex, lifestyle, hypertension, diabetes, atherosclerosis, dyslipidemia and genetic factors, are significantly associated with AMI [3–6]. However, the exact molecular mechanisms of AMI pathophysiological processes and pathologies have not been completely elucidated. With this in mind, exploring and finding hub molecules for AMI are essential to analyzing effective treatment measures and prevention methods.

Several previous studies have determined that a large fraction of noncoding RNAs (ncRNAs) have a crucial role in the modulation of biological processes and have essential functions in disease development [7, 8]. Noncoding RNAs, based on the size of the transcript, can be divided into two classes: (1) short ncRNAs (< 200 nt) that include transcription initiation RNAs, PIWI-interacting RNAs and microRNAs and (2) long ncRNAs (lncRNAs) (> 200 nt) that include natural antisense transcripts, transcribed ultraconserved regions, long intergenic ncRNAs (lincRNAs) and enhancer-like ncRNAs [9, 10]. Contrary to the short ncRNAs, which are highly conserved and their function essentially is to participate in posttranscriptional repression, lncRNAs are not well conserved, and their roles are variable [11].

**Fig.1:**
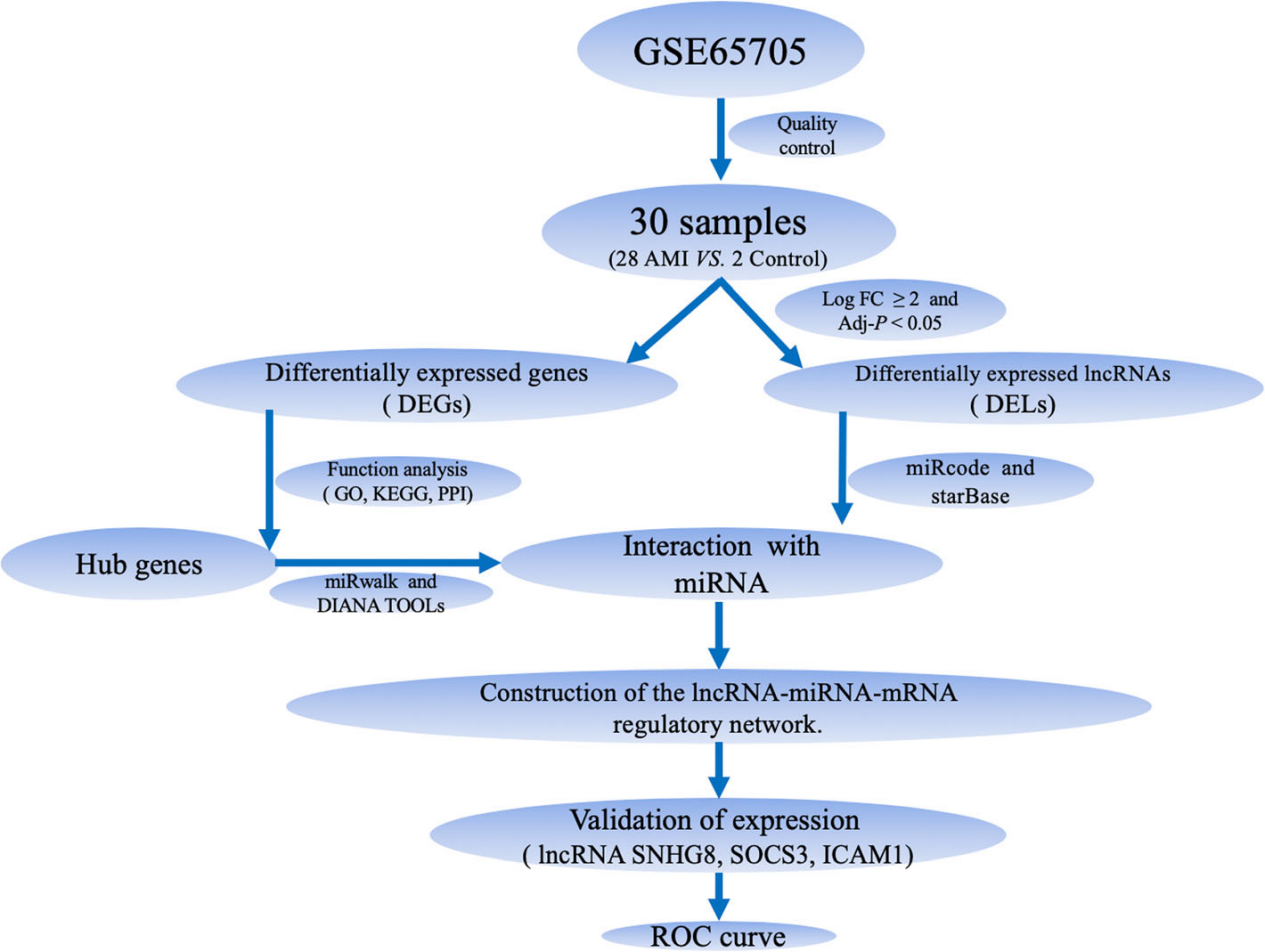
A flowchart of data analysis

Recently, increasing evidence has suggested that many ncRNAs participate in specific pathological and physiological processes of AMI [12]. As they are stable in the plasma and other body fluids, ncRNAs can regulate target mRNA translation or promote mRNA degradation. Many studies have proven that both lncRNAs and miRNAs are closely associated with the development of AMI [13]. In this study, we constructed an AMI-related lncRNA-miRNA-mRNA network by analyzing RNA-seq data and established a global triple network via the Gene Expression Omnibus (GEO) repository to explore the potential molecular mechanisms of AMI. The specific workflow is shown in Fig. 1.

## Materials and methods

### Gene expression profile probe reannotation

An RNA-seq profile dataset (GSE65705) [14] was downloaded from the Gene Expression Omnibus database (https://www.ncbi.nlm.nih.gov/geo/), which was based on the platform of GPL11154 Illumina HiSeq 2000 (*Homo sapiens*). GSE65705 contains 32 platelet samples from AMI patients (16 STEMI and 16 NSTEMI) and 2 platelet samples from normal individuals. The data analysis was performed by Reads Per Kilobase per Million mapped reads (RPKM) and quantile normalized using the robust multiarray average (RMA) method. The probes were then annotated using Bioconductor in R [15]. If one gene had more than one probe, the mean expression value of this gene was selected.

### Differentially expressed mRNAs (DEGs) and lncRNAs (DELs) and functional enrichment analysis

The limma package [16] in R was used to select the DEGs and DELs in the AMI samples compared with the normal samples. |Log_2_fold-change| ≥ 2 and adjusted *P* < 0.05 were set as the threshold for DEGs and DELs. ClusterProfiler and DOSE package in R [17] were used to perform the Gene Ontology (GO), Disease Ontology (DO) and Kyoto Encyclopedia of Genes and Genomes (KEGG) pathway analyses for DEGs. In all of the analyses, an adjusted *P*-value (Q-value) of < 0.05 was regarded as statistically significant.

### Protein-protein interaction (PPI) network construction and module analysis

The STRING database (version 11) [18] provides information on protein prediction and experimental interactions. Neighborhoods, text mining, gene fusion, databases, cooccurrence and coexpression experiments are used as the prediction methods for the database. In addition, the interactions of protein pairs in the database are presented as a combined fraction. In the current study, all DEGs were mapped to PPIs. To explore the hub genes in the network, the cutoff value was set at a combined score > 0.9 [19]. The role of protein nodes in the network was described by degrees. Network modules are one of the cores of protein networks and may have specific biological implications. The Cytoscape software package (version 3.71) was used to analyze the major clustering modules, and the most notable clustering modules were examined with Molecular Complex Detection (MCODE) [20, 21]. Subsequently, EASE ≤0.05 and a count ≥2 were set as the cutoff values and an MCODE score > 10 as the threshold for subsequent analyses.

### Prediction of miRNAs and construction of the ceRNA network

The relationships among lncRNA, miRNA, and mRNA are essential elements for the construction of the ceRNA network. We employed miRwalk and DIANA TOOLS [22, 23] to predict the targeting miRNAs for mRNAs. After GO, DO, KEGG, PPI and MCODE analyses had been completed, several meaningful DEGs were picked and mapped to targeting miRNAs. The lncRNA-miRNA interactions were predicted with the miRcode and starBase databases[24, 25], using the same mapping method as for lncRNA-miRNA interactions. Cytoscape was performed to construct and visualize the lncRNA-miRNA–mRNA ceRNA network.

### Study population

A total of 230 patients were recruited from an inpatient treatment facility for chest complaint in the Liuzhou People’s Hospital from 2018-1-1 to 2018–12-31. This study included 115 healthy participants and 115 AMI patients; the sample size was sufficient to have adequate power. The volunteers in this study were AMI patients who received treatment with percutaneous coronary intervention (PCI). (1) The inclusion criteria were as follows [26]: diagnostic criteria with reference to 2018 diagnostic guidelines for AMI patients, the elevation of cardiac biomarker (cTnT) above the upper limit of the reference value of the 99 percentile and accompanied by at least one of the following myocardial ischemia pieces of evidence: electrocardiogram revealing new ischemic changes; X-ray imaging evidence suggesting a new localized ventricular wall dysplasia or loss of viable myocardium. (2) The exclusion criteria were as follows: myocardial infarction complicated by other organ failure or serious lesions (such as lung cancer, liver and kidney failure, etc.), except for patients with allergic diseases and autoimmune diseases. The healthy control volunteers were selected by the following criteria: (1) Inclusion criteria: no history of cardiovascular disease, normal chest radiography, and normal liver and kidney function, excluding infections, tumors, etc. (2) Exclusion criteria: myocardial infarction (MI), use of thrombolytic drugs to treat cardiomyopathy, and cardiogenic shock. Clinical data were gathered for all volunteers and included baseline clinical features, angiography, and laboratory test results. For the AMI group, blood samples were the first samples obtained from patients after admission. The blood samples were collected before PCI and just a few hours after chest pain occurred, and were very suitable for the development of early diagnosis biomarkers. Clinical data collection, biochemical measurements and diagnostic criteria were performed according to previous studies [27, 28]. The Declaration of Helsinki of 1975 (http://www.wma.net/en/30publications/10policies/b3/), which was revised in 2008, was followed, and the Ethics Committee of Liuzhou people’s Hospital agreed with the study design (No: Lunshen-2017-KY; Mar. 7, 2017). Informed consent was obtained from all subjects after receiving a full explanation of the study.

### RNA isolation, reverse transcription (RT) and quantitative PCR (qPCR)

A venous blood sample of 5 ml was collected into an EDTA-coated tube from the above patients. The blood was centrifuged at 3000 g for 15 min. A NanoDrop ND-1000 spectrophotometer (NanoDrop Thermo, Wilmington, DE) was used to examine RNA quantity and quality. Subsequently, cDNA was synthesized through reverse transcription of RNA by using a reverse transcriptase kit (TIANGEN; catalog number: KR211, China). The reaction mixture included 10 μL of miRNA RT reaction buffer, 2 μL of enzyme mixture, RNase-free water (up to 20 μL) and 2 μg of total RNA. The mixture was incubated at 42 °C for 60 min, at 95 °C for 3 min, and then at 4 °C. Our quantitative RT-PCR analysis included 1 lncRNA (SNHG8) and 2 genes (SOCS3 and ICAM1), and the location and amplification of primers are shown in Additional file 1: Table S1. The primers were designed using Primer 5.0 (Shanghai Sheng Gong, China). First-strand cDNA was biosynthesized by transcribing 1 μg of tRNA using a cDNA transcription kit (thermo) with Oligo (dT) primer or random primer. Quantitative RT-PCR was performed by applying SYBR Green PCR MasterMix (Applied Biosystems, USA) with 7500 H T Fast Real-Time PCR system (Bio-Rad, USA). After the calculation of the threshold cycle (Ct) value of each sample, quantitative expression results were then obtained according to the 2^−ΔΔct^ method. PCR was performed in 10 μl reaction volumes, which included 2 μl of cDNA, 5 μl 2× Master Mix, 0.5 μl of Forward Primer (10 μM), 0.5 μl of Reverse Primer (10 μM) and 2 μl of double distilled water. The reaction was incubated at 95 °C for 15 min, at 95 °C for 28 s, at 61 °C for 30 s, and at 72 °C for 35 s. All reactions were performed in duplicate. GAPDH was used as the internal control [29].

### Statistical analysis

The statistical software package SPSS 22.0 (SPSS Inc., Chicago, IL, USA) was used in this study. A chi-square analysis was used to assess the differences in the percentages between the groups. Quantitative variables were expressed as the means ± standard deviation (TG levels are shown as medians and interquartile ranges and were analyzed by Wilcoxon-Mann-Whitney test because they were not in a normal distribution). The AMI risk score was calculated for each patient as a linear combination of selected predictors that were weighted by their respective coefficients. The ‘rms’ package was used for the AMI prediction nomogram. The predictive accuracy of the risk model was assessed by discrimination measured by C-statistic and calibration evaluated by Hosmer-Lemeshow χ^2^ statistic. To compare the plasma mRNAs and lncRNAs between the control and case groups, receiver operating characteristic (ROC) curve analysis was conducted. The diagnostic value of the mRNAs and lncRNAs was evaluated by the area under curve (AUC). All tests were two-sided, and *P* < 0.05 was considered statistically significant.

## Results

### Data preprocessing and identified differentially expressed genes

**Fig. 2:**
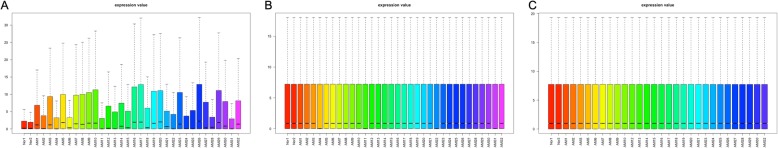
Normalization of all the samples. **a** Before normalization; (**b**) After normalization, AMI samples 4, 11, 22 and 31, which could not be normalized, were removed; (**c**) After normalization

**Fig. 3:**
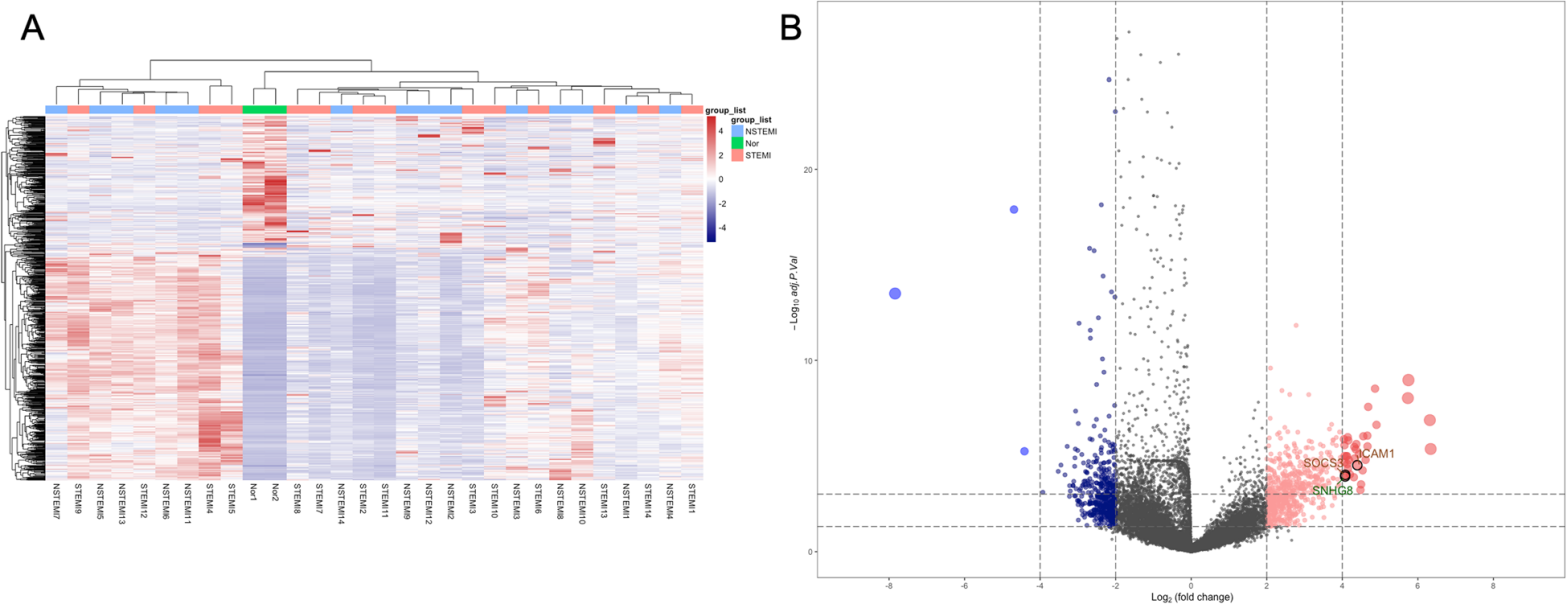
The heatmap and volcano plot of DEGs and DELs. **a** For the heatmap, the red bar represents high relative expression, and the blue bar represents low relative expression. **b** The two vertical lines are the 2-fold change boundaries, and the horizontal line is the statistical significance boundary (adjusted *P* < 0.05). Items with statistically significant up-regulation are marked with red dots, and those with significant down-regulation are marked with blue dots in the volcano plot

After quality control, we found that the AMI samples 4, 11, 22 and 31 could not be normalized (Fig. 2a), and we had to remove them from the analysis (Fig. 2b). Then, all of the rest of the samples were well normalized (Fig. 2c). Subsequently, we eliminated many incorrect expression levels and identified a total of 3127 items with adjusted *P* < 0.05 when comparing the AMI and control samples, but only identified 762 DEGs, which included 488 upregulated and 274 downregulated DEGs with |log _2_ (fold change) | ≥ 2. In addition, a total of 98 DELs, which included 55 upregulated and 43 downregulated DEGs with |log _2_ (fold change) | ≥ 2, were identified. The heatmap and volcano plot are shown in Fig. 3.

### Functional annotation, PPI network construction and identification of hub genes

**Fig. 4:**
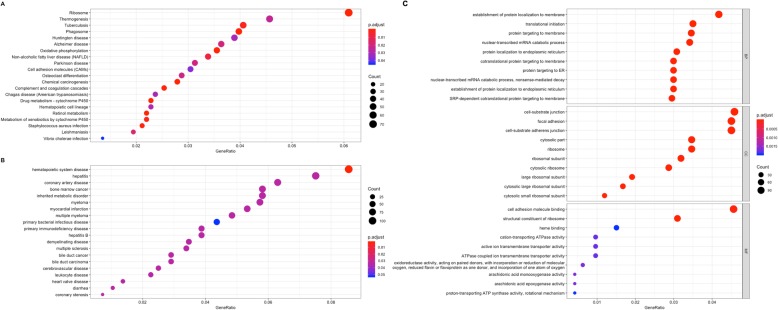
Functional annotation of DEGs. **a** KEGG analysis;(**b**) DO analysis; (**c**): GO analysis

We used the clusterProfiler package in R to carry out the KEGG pathway enrichment, DO functional and GO analyses to elucidate the role of DEGs (Fig. 4). In the analysis of GO functions, approximately 329 biological processes, 104 cellular components, and 45 molecular functions were identified with an adjusted *P* < 0.05. Table 1 shows the top 10 terms. Approximately 21 pathways were enriched in the KEGG pathway analysis and 20 DO terms were identified for the screened DEGs at adjusted *P* < 0.05 (false discovery rate, FDR set at < 0.05). Table 2 shows the top 15 items.

**Table 1:** Gene Ontology (GO) functional analysis (only the top 10 items are shown)

Items	ID	Description	*P*. adjust	geneID
BP	GO:0006614	SRP-dependent cotranslational protein targeting to membrane	7.77E-34	RPL10/SRP14/RPS28/RPS29/RPL34/RPS23/RPS24/SEC61A1/RPL21/RPS27A/RPL17/RPL39/UBA52/RPL9/RPS2/RPS10/RPL7/RPS17/RPS3/RPL31/RPL27A/RPL23A/RPL28/RPL37/RPL4/RPS18/RPL22/RPS4X/RPLP0/RPL41/RPL36/RPL14/RPL26/RPL6/RPL13/RPL13A/RPL24/RPL18A/RPS13/RPL10A/RPL7A/RPS5/RPS19/RPS6/RPS25/RPL8/RPS27/RPL29/RPL27/RPL19/RPL18/RPL38/RPS9/RPL35/RPS15A/RPL11/RPS3A/RPS21/RPS15/RPL23/SEC61B/RPLP2/RPS12/RPS14/RPS11/RPLP1/RPL30/RPS8/RPS16/RPL37A
BP	GO:0006613	cotranslational protein targeting to membrane	1.22E-33	RPL10/SRP14/RPS28/RPS29/RPL34/RPS23/RPS24/SEC61A1/RPL21/RPS27A/RPL17/RPL39/SSR2/UBA52/RPL9/RPS2/RPS10/RPL7/RPS17/RPS3/RPL31/RPL27A/RPL23A/RPL28/RPL37/RPL4/RPS18/RPL22/RPS4X/RPLP0/RPL41/RPL36/RPL14/RPL26/RPL6/RPL13/RPL13A/RPL24/RPL18A/RPS13/RPL10A/RPL7A/RPS5/RPS19/RPS6/RPS25/RPL8/RPS27/RPL29/RPL27/RPL19/RPL18/RPL38/RPS9/RPL35/RPS15A/RPL11/RPS3A/RPS21/RPS15/RPL23/SEC61B/RPLP2/RPS12/RPS14/RPS11/RPLP1/RPL30/RPS8/RPS16/RPL37A
BP	GO:0045047	protein targeting to ER	7.39E-31	RPL10/SRP14/RPS28/RPS29/RPL34/RPS23/RPS24/SEC61A1/RPL21/RPS27A/RPL17/SPCS1/RPL39/UBA52/RPL9/RPS2/RPS10/RPL7/RPS17/RPS3/RPL31/RPL27A/RPL23A/RPL28/RPL37/RPL4/RPS18/RPL22/RPS4X/RPLP0/RPL41/RPL36/RPL14/RPL26/RPL6/RPL13/RPL13A/RPL24/RPL18A/RPS13/RPL10A/RPL7A/RPS5/RPS19/RPS6/RPS25/RPL8/RPS27/RPL29/RPL27/RPL19/RPL18/RPL38/RPS9/RPL35/RPS15A/RPL11/RPS3A/RPS21/RPS15/RPL23/SEC61B/RPLP2/RPS12/RPS14/RPS11/RPLP1/RPL30/RPS8/RPS16/RPL37A
BP	GO:0000184	nuclear-transcribed mRNA catabolic process, nonsense-mediated decay	5.70E-30	MAGOHB/UPF2/SMG7/DCP1A/RPL10/RPS28/RPS29/RPL34/RPS23/RPS24/RPL21/RPS27A/RPL17/RPL39/UBA52/RPL9/RPS2/RPS10/RPL7/RPS17/RPS3/RPL31/RPL27A/RPL23A/RPL28/RPL37/RPL4/RPS18/RPL22/RPS4X/RPLP0/RPL41/RPL36/RPL14/RPL26/RPL6/RPL13/RPL13A/RPL24/RPL18A/RPS13/RPL10A/RPL7A/RPS5/RPS19/RPS6/RPS25/RPL8/RPS27/RPL29/RPL27/RPL19/RPL18/RPL38/RPS9/RPL35/RPS15A/RPL11/RPS3A/RPS21/RPS15/RPL23/RPLP2/RPS12/RPS14/RPS11/RPLP1/RPL30/RPS8/RPS16/RPL37A
BP	GO:0072599	establishment of protein localization to endoplasmic reticulum	9.67E-30	RPL10/SRP14/RPS28/RPS29/RPL34/RPS23/RPS24/SEC61A1/RPL21/RPS27A/RPL17/SPCS1/RPL39/UBA52/RPL9/RPS2/RPS10/RPL7/RPS17/RPS3/RPL31/RPL27A/RPL23A/RPL28/RPL37/RPL4/RPS18/RPL22/RPS4X/RPLP0/RPL41/RPL36/RPL14/RPL26/RPL6/RPL13/RPL13A/RPL24/RPL18A/RPS13/RPL10A/RPL7A/RPS5/RPS19/RPS6/RPS25/RPL8/RPS27/RPL29/RPL27/RPL19/RPL18/RPL38/RPS9/RPL35/RPS15A/RPL11/RPS3A/RPS21/RPS15/RPL23/SEC61B/RPLP2/RPS12/RPS14/RPS11/RPLP1/RPL30/RPS8/RPS16/RPL37A
BP	GO:0070972	protein localization to endoplasmic reticulum	1.15E-24	MIA2/RPL10/SRP14/RPS28/RPS29/RPL34/RPS23/RPS24/SEC61A1/RPL21/RPS27A/RPL17/SPCS1/RPL39/HSPA5/UBA52/RPL9/RPS2/RPS10/RPL7/RPS17/RPS3/RPL31/RPL27A/RPL23A/RPL28/RPL37/RPL4/RPS18/RPL22/RPS4X/RPLP0/RPL41/RPL36/RPL14/RPL26/RPL6/RPL13/RPL13A/RPL24/RPL18A/RPS13/RPL10A/RPL7A/RPS5/RPS19/RPS6/RPS25/RPL8/RPS27/RPL29/RPL27/RPL19/RPL18/RPL38/RPS9/RPL35/RPS15A/RPL11/RPS3A/RPS21/RPS15/RPL23/SEC61B/RPLP2/RPS12/RPS14/RPS11/RPLP1/RPL30/RPS8/RPS16/RPL37A
BP	GO:0006413	translational initiation	1.99E-22	BANK1/EIF4G3/EIF4E/FMR1/RPS6KB1/PAIP2/PAIP2B/EIF4H/RPL10/RPS28/RPS29/RPL34/EIF3F/RPS23/EIF4A2/RPS24/PPP1CA/RPL21/RPS27A/RPL17/EIF4A1/RPL39/EIF3H/UBA52/EIF3G/EIF2B5/RPL9/RPS2/RPS10/RPL7/RPS17/RPS3/RPL31/RPL27A/RPL23A/RPL28/RPL37/EIF3K/RPL4/RPS18/RPL22/RPS4X/RPLP0/RPL41/RPL36/RPL14/RPL26/RPL6/RPL13/RPL13A/RPL24/RPL18A/RPS13/RPL10A/RPL7A/RPS5/RPS19/RPS6/RPS25/RPL8/RPS27/RPL29/RPL27/RPL19/RPL18/RPL38/RPS9/RPL35/RPS15A/RPL11/RPS3A/RPS21/RPS15/RPL23/RPLP2/RPS12/RPS14/RPS11/RPLP1/RPL30/RPS8/RPS16/RPL37A
BP	GO:0006612	protein targeting to membrane	4.64E-21	ZDHHC15/ATG4C/CHM/CEMIP/RABGEF1/RAB3IP/TCAF1/C2CD5/RTP3/PRNP/RPL10/SRP14/RPS28/RPS29/RPL34/RPS23/RPS24/SEC61A1/RPL21/RPS27A/RPL17/RPL39/SSR2/UBA52/RPL9/RPS2/RPS10/RPL7/RPS17/RPS3/RPL31/RPL27A/RPL23A/RPL28/RPL37/RPL4/RPS18/RPL22/RPS4X/RPLP0/RPL41/RPL36/RPL14/RPL26/RPL6/RPL13/RPL13A/RPL24/RPL18A/RPS13/RPL10A/RPL7A/RPS5/RPS19/RPS6/RPS25/RPL8/RPS27/RPL29/RPL27/RPL19/RPL18/RPL38/RPS9/RPL35/RPS15A/RPL11/RPS3A/RPS21/RPS15/RPL23/SEC61B/RPLP2/RPS12/RPS14/RPS11/RPLP1/RPL30/RPS8/RPS16/ITGB2/RPL37A
BP	GO:0000956	nuclear-transcribed mRNA catabolic process	6.09E-19	CNOT4/MAGOHB/CNOT11/PAN3/XRN1/RC3H1/TTC37/UPF2/SMG7/DCP1A/RPL10/LSM4/RPS28/RPS29/RPL34/RPS23/RPS24/RPL21/RPS27A/RPL17/RPL39/UBA52/RPL9/RPS2/RPS10/RPL7/RPS17/RPS3/RPL31/RPL27A/ZFP36/RPL23A/RPL28/RPL37/RPL4/RPS18/RPL22/RPS4X/RPLP0/RPL41/LSM7/RPL36/RPL14/RPL26/RPL6/RPL13/RPL13A/RPL24/RPL18A/ZFP36L2/RPS13/RPL10A/RPL7A/RPS5/RPS19/RPS6/RPS25/RPL8/RPS27/RPL29/RPL27/RPL19/RPL18/RPL38/RPS9/RPL35/RPS15A/RPL11/RPS3A/RPS21/RPS15/RPL23/RPLP2/RPS12/RPS14/RPS11/RPLP1/RPL30/RPS8/RPS16/RPL37A
BP	GO:0090150	establishment of protein localization to membrane	2.80E-15	RILPL1/ZDHHC15/CNST/ATG4C/BRAF/CLIP1/BLZF1/CHM/CEMIP/SNAP25/RABGEF1/TP53BP2/YWHAE/RAB3IP/TCAF1/RAB31/C2CD5/RTP3/EGFR/ANXA13/CALM1/PRNP/RPL10/SRP14/RPS28/YWHAB/RPS29/RPL34/RPS23/CLSTN1/RPS24/SEC61A1/RPL21/RPS27A/RPL17/RPL39/SSR2/UBA52/RPL9/RPS2/RPS10/RPL7/RPS17/RPS3/RPL31/RPL27A/RPL23A/RPL28/RPL37/RPL4/RPS18/RPL22/RPS4X/RPLP0/RPL41/NDUFA13/RPL36/RPL14/RPL26/RPL6/RPL13/RPL13A/RPL24/RPL18A/RPS13/RPL10A/RPL7A/RPS5/RPS19/RPS6/RPS25/RPL8/RPS27/RPL29/RACK1/RPL27/RPL19/RPL18/RPL38/RPS9/RPL35/RPS15A/GZMB/RPL11/RPS3A/RPS21/RPS15/RPL23/SEC61B/RPLP2/RPS12/RPS14/RPS11/RPLP1/RPL30/RPS8/RPS16/ITGB2/RPL37A
CC	GO:0022626	cytosolic ribosome	3.57E-33	LARP4/NAA11/RPL10/RPS28/RPS29/RPL34/ZNF622/RPS23/RPS24/RPL21/RPS27A/RPL17/RPL39/UBA52/RPL9/RPS2/RPS10/RPL7/RPS17/RPS3/RPL31/RPL27A/RPL23A/RPL28/RPL37/RPL4/RPS18/RPL22/RPS4X/RPLP0/RPL41/RPL36/RPL14/RPL26/RPL6/RPL13/RPL13A/RPL24/RPL18A/RPS13/RPL10A/RPL7A/RPS5/RPS19/RPS6/RPL22L1/RPS25/RPL8/RPS27/RPL29/RACK1/RPL27/RPL19/RPL18/RPL38/RPS9/RPL35/RPS15A/RPL11/RPS3A/RPS21/RPS15/RPL23/RPLP2/RPS12/RPS14/RPS11/RPLP1/RPL30/RPS8/RPS16/RPL37A
CC	GO:0044391	ribosomal subunit	2.57E-21	MRPL30/LARP4/RPL10/MRPS35/MRPS21/RPS28/RPS29/RPL34/ZNF622/RPS23/RBM3/RPS24/RPL21/MRPL51/RPS27A/RPL17/RPL39/UBA52/RPL9/RPS2/RPS10/MRPS24/MRPL16/RPL7/RPS17/RPS3/RPL31/RPL27A/RPL23A/RPL28/RPL37/RPL4/RPS18/RPL22/RPS4X/RPLP0/RPL41/RPL36/MRPL55/RPL14/RPL26/RPL6/RPL13/RPL13A/RPL24/RPL18A/RPS13/RPL10A/RPL7A/RPS5/RPS19/RPS6/RPL22L1/RPS25/RPL8/RPS27/RPL29/RACK1/RPL27/RPL19/RPL18/RPL38/RPS9/RPL35/RPS15A/RPL11/RPS3A/RPS21/RPS15/RPL23/RPLP2/RPS12/RPS14/NDUFAB1/RPS11/RPLP1/RPL30/RPS8/RPS16/RPL37A
CC	GO:0022625	cytosolic large ribosomal subunit	8.94E-21	RPL10/RPL34/ZNF622/RPL21/RPL17/RPL39/UBA52/RPL9/RPL7/RPL31/RPL27A/RPL23A/RPL28/RPL37/RPL4/RPL22/RPLP0/RPL41/RPL36/RPL14/RPL26/RPL6/RPL13/RPL13A/RPL24/RPL18A/RPL10A/RPL7A/RPL22L1/RPL8/RPL29/RPL27/RPL19/RPL18/RPL38/RPL35/RPL11/RPL23/RPLP2/RPLP1/RPL30/RPL37A
CC	GO:0044445	cytosolic part	3.38E-17	HOMER1/STXBP5/EIF4E/NEDD4/LARP4/SNAP25/ERC1/TRIM9/PFKFB1/NAA11/RPL10/CASP4/ENO1/NLRP1/PSMC5/RPS28/RPS29/RPL34/ZNF622/RPS23/RPS24/RPL21/RPS27A/NLRC4/RPL17/RPL39/UBA52/RPL9/RPS2/RPS10/RPL7/RPS17/RPS3/RPL31/RPL27A/RPL23A/RPL28/RPL37/RPL4/RPS18/RPL22/GSDMD/RPS4X/RPLP0/RPL41/RPL36/RPL14/RPL26/RPL6/RPL13/RPL13A/RPL24/RPL18A/RPS13/RPL10A/RPL7A/RPS5/RPS19/RPS6/RPL22L1/RPS25/RPL8/RPS27/RPL29/RACK1/RPL27/RPL19/RPL18/RPL38/RPS9/RPL35/RPS15A/RPL11/RPS3A/RPS21/RPS15/RPL23/PYCARD/RPLP2/RPS12/RPS14/RPS11/RPLP1/RPL30/RPS8/RPS16/RPL37A
CC	GO:0030055	cell-substrate junction	7.50E-15	RDX/PPP1R12A/ITGA1/SORBS2/NEXN/LPP/LMLN/XIRP2/ADAM9/EFNB2/DAB2/FOCAD/SENP1/DCAF6/DLC1/SYNPO2/PTPN12/DMD/YWHAE/ARHGEF7/LIMS1/DIXDC1/ERBIN/KRAS/ITGBL1/AKAP12/ITGA11/CBL/DDR2/BCAR1/SPRY4/NFASC/THY1/EGFR/ITGA8/S100A7/NOX4/RHOA/LASP1/ARF1/RHOG/ATP6V0C/PFN1/ACTB/CNN2/ARPC2/CFL1/YWHAB/RPS29/ACTG1/SCARB2/GAK/PPIA/EVL/FES/PLEC/IQGAP1/RHOB/AHNAK/HSPA5/HSP90B1/RPL9/RPS2/VIM/ICAM1/RPS10/RPL7/RPS17/RPS3/PTPRC/RPL31/HCK/ARPC3/LRP1/ITGB7/CD81/PARVG/RPL4/ANXA1/RAC2/RPS18/RPL22/IGF2R/RPS4X/CALR/RPLP0/ITGA4/RPL6/RPL13A/RPS13/RPL10A/RPL7A/RPS5/RPS19/RPL8/RPL27/RPL19/RPL18/RPL38/RPS9/PPIB/RPS3A/RPS15/RPL23/PLAUR/RPLP2/RPS14/RPS11/RPLP1/RPL30/RPS8/RPS16/ITGB2/RPL37A/CYBA
CC	GO:0022627	cytosolic small ribosomal subunit	7.70E-15	LARP4/RPS28/RPS29/RPS23/RPS24/RPS27A/UBA52/RPS2/RPS10/RPS17/RPS3/RPS18/RPS4X/RPS13/RPS5/RPS19/RPS6/RPS25/RPS27/RACK1/RPS9/RPS15A/RPS3A/RPS21/RPS15/RPS12/RPS14/RPS11/RPS8/RPS16
CC	GO:0005925	focal adhesion	1.03E-14	RDX/PPP1R12A/ITGA1/SORBS2/NEXN/LPP/LMLN/XIRP2/ADAM9/EFNB2/DAB2/FOCAD/SENP1/DCAF6/DLC1/SYNPO2/PTPN12/YWHAE/ARHGEF7/LIMS1/DIXDC1/KRAS/ITGBL1/AKAP12/ITGA11/CBL/DDR2/BCAR1/SPRY4/NFASC/THY1/EGFR/ITGA8/S100A7/NOX4/RHOA/LASP1/ARF1/RHOG/ATP6V0C/PFN1/ACTB/CNN2/ARPC2/CFL1/YWHAB/RPS29/ACTG1/SCARB2/GAK/PPIA/EVL/FES/PLEC/IQGAP1/RHOB/AHNAK/HSPA5/HSP90B1/RPL9/RPS2/VIM/ICAM1/RPS10/RPL7/RPS17/RPS3/PTPRC/RPL31/HCK/ARPC3/LRP1/ITGB7/CD81/PARVG/RPL4/ANXA1/RAC2/RPS18/RPL22/IGF2R/RPS4X/CALR/RPLP0/ITGA4/RPL6/RPL13A/RPS13/RPL10A/RPL7A/RPS5/RPS19/RPL8/RPL27/RPL19/RPL18/RPL38/RPS9/PPIB/RPS3A/RPS15/RPL23/PLAUR/RPLP2/RPS14/RPS11/RPLP1/RPL30/RPS8/RPS16/ITGB2/RPL37A/CYBA
CC	GO:0005924	cell-substrate adherens junction	1.63E-14	RDX/PPP1R12A/ITGA1/SORBS2/NEXN/LPP/LMLN/XIRP2/ADAM9/EFNB2/DAB2/FOCAD/SENP1/DCAF6/DLC1/SYNPO2/PTPN12/YWHAE/ARHGEF7/LIMS1/DIXDC1/KRAS/ITGBL1/AKAP12/ITGA11/CBL/DDR2/BCAR1/SPRY4/NFASC/THY1/EGFR/ITGA8/S100A7/NOX4/RHOA/LASP1/ARF1/RHOG/ATP6V0C/PFN1/ACTB/CNN2/ARPC2/CFL1/YWHAB/RPS29/ACTG1/SCARB2/GAK/PPIA/EVL/FES/PLEC/IQGAP1/RHOB/AHNAK/HSPA5/HSP90B1/RPL9/RPS2/VIM/ICAM1/RPS10/RPL7/RPS17/RPS3/PTPRC/RPL31/HCK/ARPC3/LRP1/ITGB7/CD81/PARVG/RPL4/ANXA1/RAC2/RPS18/RPL22/IGF2R/RPS4X/CALR/RPLP0/ITGA4/RPL6/RPL13A/RPS13/RPL10A/RPL7A/RPS5/RPS19/RPL8/RPL27/RPL19/RPL18/RPL38/RPS9/PPIB/RPS3A/RPS15/RPL23/PLAUR/RPLP2/RPS14/RPS11/RPLP1/RPL30/RPS8/RPS16/ITGB2/RPL37A/CYBA
CC	GO:0005840	ribosome	1.68E-14	MRPL30/LARP4/FMR1/DNAJC21/NAA11/RPL10/MRPS35/MRPS21/RPS28/RPS29/RPL34/ZNF622/RPS23/RBM3/AURKAIP1/RPS24/RPL21/MRPL51/RPS27A/RPL17/RPL39/EIF3H/METTL17/UBA52/RPL9/RPS2/RPS10/MRPS24/MRPL16/RPL7/RPS17/RPS3/RPL31/RPL27A/EEF2/RPL23A/RPL28/RPL37/RPL4/RPS18/RPL22/RPS4X/RPLP0/RPL41/RPL36/MRPL55/RPL14/RPL26/RPL6/RPL13/RPL13A/RPL24/RPL18A/RPS13/RPL10A/RPL7A/RPS5/RPS19/RPS6/RPL22L1/RPS25/RPL8/RPS27/RPL29/RACK1/RPL27/RPL19/RPL18/RPL38/RPS9/RPL35/RPS15A/RPL11/RPS3A/RPS21/RPS15/RPL23/RPLP2/RPS12/RPS14/NDUFAB1/RPS11/RPLP1/RPL30/RPS8/RPS16/RPL37A
CC	GO:0015934	large ribosomal subunit	6.10E-12	MRPL30/RPL10/RPL34/ZNF622/RBM3/RPL21/MRPL51/RPL17/RPL39/UBA52/RPL9/MRPL16/RPL7/RPL31/RPL27A/RPL23A/RPL28/RPL37/RPL4/RPL22/RPLP0/RPL41/RPL36/MRPL55/RPL14/RPL26/RPL6/RPL13/RPL13A/RPL24/RPL18A/RPL10A/RPL7A/RPL22L1/RPL8/RPL29/RPL27/RPL19/RPL18/RPL38/RPL35/RPL11/RPL23/RPLP2/NDUFAB1/RPLP1/RPL30/RPL37A
MF	GO:0003735	structural constituent of ribosome	5.22E-14	MRPL30/RPL10/MRPS35/MRPS21/RPS28/RPS29/RPL34/RPS23/RPS24/RPL21/MRPL51/RPS27A/RPL17/RPL39/UBA52/RPL9/RPS2/RPS10/MRPS24/MRPL16/RPL7/RPS17/RPS3/RPL31/RPL27A/RPL23A/RPL28/RPL37/RPL4/RPS18/RPL22/RPS4X/RPLP0/RPL41/RPL36/MRPL55/RPL14/RPL26/RPL6/RPL13/RPL13A/RPL24/RPL18A/RPS13/RPL10A/RPL7A/RPS5/RPS19/RPS6/RPL22L1/RPL8/RPS27/RPL29/RPL27/RPL19/RPL18/RPL38/RPS9/RPL35/RPS15A/RPL11/RPS3A/RPS21/RPS15/RPL23/RPLP2/RPS12/RPS14/RPS11/RPLP1/RPL30/RPS8/RPS16/RPL37A
MF	GO:0050839	cell adhesion molecule binding	2.33E-05	ANLN/NLGN1/TJP1/FGA/PDLIM5/RDX/FGF2/ASAP1/GOLGA2/CTNND2/CTNNA3/FGG/CD2AP/ADAM9/DLG1/CIP2A/CDH20/COBLL1/FGB/ABI1/UBAP2/FN1/USP8/PKN2/ERC1/MYO1B/HMGB1/LRRFIP1/YWHAE/VTN/SLK/PTPRD/MRTFB/COL3A1/CKAP5/LGALS8/SFRP2/CXCL12/LRRC4C/ITGBL1/CTGF/CLINT1/CBL/PROM1/PTPN11/EMP2/WISP1/CDH19/TSPAN8/CYR61/EDIL3/NPNT/THY1/EGFR/ADAMTS8/CDHR2/TNN/EVPL/LASP1/EIF4H/ICAM2/FXYD5/PFN1/CNN2/EFHD2/ENO1/CLIC1/DNAJB1/YWHAB/ALDOA/RPL34/CAPZB/PPP1CA/RAN/PRDX1/EEF1G/P2RX4/PLEC/NOP56/IQGAP1/AHNAK/ANXA2/HSPA5/CEMIP2/RPS2/ICAM1/EEF2/EEF1D/ITGB7/RPL23A/ADAM8/CD81/ANXA1/H1FX/PSMB6/CD1D/CALR/ITGAL/ITGA4/S100A11/JAML/RPL14/LILRB2/RPL6/RPL24/RPL7A/RPL29/RACK1/ITGB2
MF	GO:0016712	oxidoreductase activity, acting on paired donors, with incorporation or reduction of molecular oxygen, reduced flavin or flavoprotein as one donor, and incorporation of one atom of oxygen	1.55E-03	CYP2E1/CYP2C8/CYP3A4/CYP19A1/CYP3A5/CYP2C9/CYP2A6/CYP2C19/CYP2B6/CYP4F12/CYP2C18/CYP2A7/CYP1A2/CYP3A43/CYP1B1
MF	GO:0008391	arachidonic acid monooxygenase activity	1.55E-03	CYP2E1/CYP2C8/CYP4A11/CYP2C9/CYP2A6/CYP2C19/CYP2B6/CYP4F12/CYP2C18/CYP2A7
MF	GO:0008392	arachidonic acid epoxygenase activity	1.55E-03	CYP2E1/CYP2C8/CYP4A11/CYP2C9/CYP2A6/CYP2C19/CYP2B6/CYP4F12/CYP2C18/CYP2A7
MF	GO:0019829	cation-transporting ATPase activity	1.63E-03	ATP13A5/ATP7A/ATP8A1/ATP6V1C1/ATP2B2/ABCB11/ATP5MG/ATP6V0C/ATP6AP1/ATP6V1B2/ATP2B4/ATP6V1F/ATP2B1/ATP5F1B/ATP5F1E/ATP5F1A/ATP5PD/ATP5MC2/ATP5PO/ATP5F1D/ATP1B3/ATP6V0B/TCIRG1
MF	GO:0022853	active ion transmembrane transporter activity	1.63E-03	ATP13A5/ATP7A/ATP8A1/ATP6V1C1/ATP2B2/ABCB11/ATP5MG/ATP6V0C/ATP6AP1/ATP6V1B2/ATP2B4/ATP6V1F/ATP2B1/ATP5F1B/ATP5F1E/ATP5F1A/ATP5PD/ATP5MC2/ATP5PO/ATP5F1D/ATP1B3/ATP6V0B/TCIRG1
MF	GO:0042625	ATPase coupled ion transmembrane transporter activity	1.63E-03	ATP13A5/ATP7A/ATP8A1/ATP6V1C1/ATP2B2/ABCB11/ATP5MG/ATP6V0C/ATP6AP1/ATP6V1B2/ATP2B4/ATP6V1F/ATP2B1/ATP5F1B/ATP5F1E/ATP5F1A/ATP5PD/ATP5MC2/ATP5PO/ATP5F1D/ATP1B3/ATP6V0B/TCIRG1
MF	GO:0046933	proton-transporting ATP synthase activity, rotational mechanism	1.82E-03	ATP5MG/ATP6V0C/ATP6AP1/ATP5F1B/ATP5F1E/ATP5F1A/ATP5PD/ATP5MC2/ATP5PO/ATP5F1D
MF	GO:0020037	heme binding	1.82E-03	CYP2E1/CYP2C8/HRG/CYP3A4/AMBP/FLVCR1/CYP19A1/CYP3A5/CYP4A11/ADGB/CYP2C9/CYP2A6/CYP2C19/CYP2B6/CYP8B1/PXDN/CYP4F12/CYP2C18/PTGIS/CYP2A7/CYP4F11/CYP1A2/CYP7A1/CYP3A43/MB/NOS1/NOX4/CYP4A22/SDHC/SDHD/CYP4V2/CYB561D2/CYC1/CYP1B1/CYBB/CYBA

*BP* biological processes, *CC* cellular components, *MF* molecular functions

**Table 2:** Kyoto Encyclopedia of Genes and Genomes (KEGG) pathway and disease ontology (DO) analyses (only the top 15 items are shown)

Items	ID	Description	*P*. adjust	geneID
KEGG	hsa03010	Ribosome	8.44E-18	MRPL30/RPL10/MRPS21/RPS28/RPS29/RPL34/RPS23/RPS24/RPL21/RPS27A/RPL17/RPL39/UBA52/RPL9/RPS2/RPS10/MRPL16/RPL7/RPS17/RPS3/RPL31/RPL27A/RPL23A/RPL28/RPL37/RPL4/RPS18/RPL22/RPS4X/RPLP0/RPL41/RPL36/RPL14/RPL26/RPL6/RPL13/RPL13A/RPL24/RPL18A/RPS13/RPL10A/RPL7A/RPS5/RPS19/RPS6/RPL22L1/RPS25/RPL8/RPS27/RPL29/RPL27/RPL19/RPL18/RPL38/RPS9/RPL35/RPS15A/RPL11/RPS3A/RPS21/RPS15/RPL23/FAU/RPLP2/RPS12/RPS14/RPS11/RPLP1/RPL30/RPS8/RPS16/RPL37A
KEGG	hsa05204	Chemical carcinogenesis	1.04E-05	CYP2E1/CYP2C8/ADH6/CYP3A4/UGT2B28/ADH4/ADH1B/CYP3A5/ADH1A/CYP2C9/UGT2B11/CYP2A6/ADH1C/CYP2C19/SULT2A1/UGT1A6/CYP2C18/UGT2A3/UGT2B15/UGT2A1/UGT2B4/HSD11B1/UGT1A1/CYP1A2/CYP3A43/GSTO1/SULT1A1/GSTK1/CYP1B1/SULT1A2/SULT1A4/SULT1A3/GSTP1
KEGG	hsa04610	Complement and coagulation cascades	1.20E-04	FGA/F8/FGG/C3/C7/FGB/F2R/KNG1/VTN/C1S/PLG/CFB/SERPINC1/C1R/F9/CFI/C9/C6/SERPINA5/MASP1/F7/C8A/C4BPA/F11/MBL2/BDKRB2/C5AR1/SERPINA1/PLAUR/ITGB2
KEGG	hsa04145	Phagosome	1.20E-04	C3/EEA1/TUBAL3/STX7/TUBB1/ATP6V1C1/RAB7A/TUBB4A/C1R/COLEC11/CLEC4M/MBL2/NOS1/LAMP1/ATP6V0C/ATP6AP1/HLA-C/ACTB/TUBA1A/ATP6V1B2/ATP6V1F/TUBA1B/ACTG1/SEC61A1/TAP2/TLR4/NCF2/HLA-DMA/NCF4/CALR/TLR2/CLEC7A/FCGR3A/ATP6V0B/CORO1A/CYBB/HLA-DRB1/HLA-DPA1/TCIRG1/HLA-DRB5/HLA-DMB/SEC61B/HLA-DRA/CD14/CTSS/ITGB2/CYBA
KEGG	hsa00190	Oxidative phosphorylation	1.89E-04	ATP6V1C1/COX6B2/UQCRH/ATP5MG/ATP6V0C/NDUFA2/ATP6AP1/SDHC/UQCR10/ATP6V1B2/ATP6V1F/NDUFB4/SDHD/NDUFA1/NDUFB11/ATP5F1B/NDUFC2/UQCRQ/NDUFA11/NDUFB9/COX4I1/ATP5F1E/COX5A/ATP5F1A/ATP5PD/ATP5MC2/UQCRC1/NDUFB10/CYC1/ATP5ME/UQCR11/COX8A/ATP5PO/ATP5F1D/NDUFA13/ATP6V0B/ATP5MF/COX7C/NDUFB2/TCIRG1/COX5B/NDUFAB1
KEGG	hsa00830	Retinol metabolism	1.93E-04	CYP2C8/ALDH1A2/ADH6/CYP3A4/UGT2B28/ADH4/ADH1B/CYP3A5/CYP4A11/ADH1A/CYP2C9/UGT2B11/CYP2A6/ADH1C/CYP2B6/UGT1A6/CYP2C18/UGT2A3/UGT2B15/UGT2A1/UGT2B4/UGT1A1/CYP1A2/AOX1/CYP4A22/DGAT1
KEGG	hsa00982	Drug metabolism - cytochrome P450	2.32E-04	CYP2E1/CYP2C8/ADH6/CYP3A4/UGT2B28/ADH4/ADH1B/CYP3A5/ADH1A/CYP2C9/UGT2B11/CYP2A6/ADH1C/CYP2C19/CYP2B6/FMO3/UGT1A6/UGT2A3/UGT2B15/UGT2A1/UGT2B4/UGT1A1/CYP1A2/AOX1/GSTO1/GSTK1/GSTP1
KEGG	hsa05150	*Staphylococcus aureus* infection	6.74E-04	FGG/C3/C1S/PLG/CFB/C1R/CFI/MASP1/DEFA5/DEFB1/MBL2/ICAM1/HLA-DMA/FPR2/ITGAL/FCGR3A/HLA-DRB1/C5AR1/HLA-DPA1/HLA-DRB5/FPR1/SELPLG/HLA-DMB/HLA-DRA/ITGB2
KEGG	hsa00980	Metabolism of xenobiotics by cytochrome P450	1.75E-03	CYP2E1/ADH6/CYP3A4/UGT2B28/ADH4/ADH1B/CYP3A5/ADH1A/CYP2C9/UGT2B11/CYP2A6/ADH1C/CYP2B6/SULT2A1/UGT1A6/UGT2A3/UGT2B15/UGT2A1/UGT2B4/HSD11B1/UGT1A1/CYP1A2/GSTO1/GSTK1/CYP1B1/GSTP1
KEGG	hsa05152	Tuberculosis	2.52E-03	TGFB2/MAPK10/CAMK2D/C3/EEA1/AKT3/NFYB/RAB7A/MAPK9/IFNA16/EP300/IFNB1/IFNA4/CLEC4M/LBP/CALM1/RHOA/LAMP1/ATP6V0C/ATP6AP1/MYD88/IRAK1/IFNGR2/TLR4/NOD2/CEBPB/CTSD/FCER1G/IL10RB/HLA-DMA/TNFRSF1A/TLR1/TLR2/CLEC7A/FCGR3A/ATP6V0B/CORO1A/HLA-DRB1/HLA-DPA1/CD74/TCIRG1/IL10RA/HLA-DRB5/HLA-DMB/HLA-DRA/CD14/CTSS/ITGB2
KEGG	hsa04932	Non-alcoholic fatty liver disease (NAFLD)	8.56E-03	MAPK10/CYP2E1/PIK3R3/AKT3/ADIPOR1/GSK3B/PIK3CA/MAPK9/PRKAA1/ADIPOQ/MLXIPL/COX6B2/UQCRH/NDUFA2/SDHC/UQCR10/NDUFB4/SDHD/NDUFA1/IL6R/NDUFB11/NDUFC2/UQCRQ/NDUFA11/NDUFB9/COX4I1/COX5A/TNFRSF1A/UQCRC1/NDUFB10/CYC1/UQCR11/COX8A/NDUFA13/XBP1/COX7C/NDUFB2/COX5B/NDUFAB1/SOCS3
KEGG	hsa05140	Leishmaniasis	1.70E-02	TGFB2/C3/TAB2/MYD88/IRAK1/IFNGR2/TLR4/NCF2/HLA-DMA/NCF4/TLR2/ITGA4/FCGR3A/CYBB/HLA-DRB1/HLA-DPA1/NFKBIA/HLA-DRB5/HLA-DMB/HLA-DRA/FOS/ITGB2/CYBA
KEGG	hsa05010	Alzheimer disease	1.98E-02	PLCB4/GNAQ/ITPR2/CACNA1F/GSK3B/APOE/COX6B2/NOS1/CALM1/UQCRH/NDUFA2/SDHC/UQCR10/NDUFB4/SDHD/NDUFA1/NDUFB11/ATP5F1B/NDUFC2/UQCRQ/NDUFA11/NDUFB9/COX4I1/ATP5F1E/GAPDH/COX5A/ATP5F1A/ATP5PD/ATP5MC2/TNFRSF1A/UQCRC1/NDUFB10/LRP1/CYC1/UQCR11/COX8A/ATP5PO/ATP5F1D/NDUFA13/COX7C/NDUFB2/COX5B/NDUFAB1
KEGG	hsa05012	Parkinson disease	2.06E-02	PRKACB/COX6B2/UQCRH/GNAL/NDUFA2/SDHC/UQCR10/SLC25A5/NDUFB4/SDHD/NDUFA1/UBB/NDUFB11/ATP5F1B/NDUFC2/UQCRQ/NDUFA11/NDUFB9/COX4I1/ATP5F1E/COX5A/ATP5F1A/ATP5PD/ATP5MC2/UQCRC1/NDUFB10/CYC1/UQCR11/COX8A/ATP5PO/ATP5F1D/SLC25A6/NDUFA13/COX7C/NDUFB2/COX5B/NDUFAB1
KEGG	hsa04380	Osteoclast differentiation	2.12E-02	TGFB2/MAPK10/PIK3R3/AKT3/TAB2/PIK3CA/MAPK9/IFNB1/TNFRSF11B/GRB2/JUND/TGFBR1/IFNAR1/LILRB1/STAT2/IFNGR2/LILRA2/NCF2/SIRPA/LILRA6/TNFRSF1A/NCF4/LILRB2/FCGR3A/OSCAR/NFKBIA/LILRB3/LILRA5/SOCS3/CSF1R/FOS/TYROBP/CYBA/JUNB
DO	DOID:74	hematopoietic system disease	1.74E-04	ALB/FGA/CYP2C8/FGF2/HFE/APOB/FGG/C3/APOC3/APOA1/ADH1B/BDNF/APOH/FGB/FN1/F2R/TUBB1/LRRFIP1/CYP2C9/PON1/PLG/CXCL12/SERPINC1/FABP3/ADH1C/CYP2C19/CCL2/PAPPA/IGFBP1/MEF2A/APOE/PROM1/AHSG/F7/PTGIS/CCL11/ABCC9/UGT1A1/CYP1A2/ADIPOQ/INHBA/F11/CRP/TNFRSF11B/MB/MBL2/TNNT2/ELN/PPIA/TLR4/ALOX5/CTSD/ICAM1/PTPRC/ALOX5AP/ADAM8/GPX1/TLR2/PRF1/CST3/GZMB/PLAUR/CD14/CXCR4/TNFRSF1B/S100A9
DO	DOID:5844	myocardial infarction	2.06E-04	KITLG/NFAT5/FGF2/TET2/BDNF/FIP1L1/IL5/KRAS/CCL11/UGT1A1/IL25/CFTR/NTRK3/MBL2/RASGRP4/TLR4/HAX1/TNFRSF1A/CD8A/ITPA/TLR8/FCGR3A/CSF3R/CXCR3/CD4/RNASE2/CXCR4/DUSP1
DO	DOID:9500	leukocyte disease	2.97E-04	ALB/FGA/CYP2C8/CDKN2B/FGF2/HFE/APOB/FGG/C3/APOC1/APOC3/CYP3A4/APOA1/ADH1B/BDNF/APOC2/APOH/FGB/FN1/F2R/SPP1/TUBB1/LRRFIP1/CYP2C9/MTR/PON1/PLG/CXCL12/SERPINC1/FABP3/ADH1C/CYP2C19/CCL2/PAPPA/IGFBP1/MEF2A/APOE/PROM1/AHSG/F7/PTGIS/CCL11/ABCC9/UGT1A1/CYP1A2/ADIPOQ/INHBA/F11/CRP/TFAP2B/TNFRSF11B/NPC1L1/MB/MBL2/TNNT2/ELN/CNDP1/PPIA/TLR4/ALOX5/CTSD/ICAM1/PTPRC/ALOX5AP/ADAM8/GPX1/TLR2/ITGAL/PRF1/CST3/GZMB/SELPLG/PLAUR/CD14/CXCR4/TNFRSF1B/S100A9/CYBA
DO	DOID:3393	coronary artery disease	3.32E-04	AR/PTBP2/RBP4/CYP2E1/TJP1/CCL21/FGF2/HFE/FGG/APOC1/APOC3/VRK1/AMBP/MKI67/APOA1/NEDD4/APOH/FGB/ADIPOR1/DLC1/F2R/SPP1/HMGB1/DNMT1/TIA1/CYP2A6/GSK3B/PIK3CA/PON1/CXCL12/CCL2/KRAS/NR5A2/TCEA1/CBL/IFNB1/APOE/AHSG/HNF4A/UGT1A6/OPRD1/ADIPOQ/EGFR/CLEC4M/MBL2/CAPNS1/ARF1/GRB2/TMSB4X/HLAC/VIPR1/MYD88/DNAJB1/IFNAR1/IL6R/PPIA/TLR4/ALOX5/PSMB9/OAS1/CDKN1B/EIF4A1/LAMTOR5/HSPA5/VIM/ICAM1/TNFRSF1A/IRF1/PTPRC/TNFSF10/CD81/REX1BD/TLR2/FGL2/DGAT1/ITGAL/PRF1/CCL4/FCGR3A/HLA-DRB1/CXCR3/HLA-DPA1/SERPINA1/IL10RA/GZMB/GSTP1/PLAUR/CD4/CD14/SOCS3/FCN2/CXCR4/TNFRSF1B
DO	DOID:2237	hepatitis	5.74E-04	NFAT5/CCL21/LIG4/C3/APOA1/MLLT3/DNMT3B/PRKAR1A/HEXIM1/FOXO3/CXCL12/CYP2B6/IL5/PTN/FOXN1/APOE/PROM1/HIF3A/UGT1A1/BMP2/TNFRSF11B/NGF/MBL2/CTSC/PPIA/NCF2/CD63/TAPBP/ICOS/NCF4/CD86/TLR2/CD3E/PRF1/SRSF5/FCGR3A/CYBB/HLA-DRB1/BST2/SERPINA1/FCGRT/PLAUR/CD4/CXCR4/CD3D/TNFRSF1B/ITGB2/CYBA
DO	DOID:612	primary immunodeficiency disease	6.33E-04	SPP1/CCL2/APOE/CRP/TLR4/ICAM1/TLR2/ITGAL/CYBA
DO	DOID:4248	coronary stenosis	6.49E-04	AR/HFE/FGG/APOC3/APOA1/NEDD4/APOH/DLC1/HMGB1/DNMT1/TIA1/GSK3B/CCL2/KRAS/NR5A2/IFNB1/APOE/AHSG/HNF4A/ADIPOQ/MBL2/CAPNS1/TMSB4X/HLA-C/MYD88/DNAJB1/IFNAR1/IL6R/TLR4/ALOX5/PSMB9/OAS1/LAMTOR5/HSPA5/TNFRSF1A/TNFSF10/REX1BD/TLR2/FGL2/PRF1/HLA-DRB1/HLA-DPA1/GZMB/GSTP1/PLAUR/CD14/SOCS3/CXCR4
DO	DOID:2043	hepatitis B	9.04E-04	ITGA1/ATXN2/HFE/C3/C7/MBP/EBF1/APOA1/BDNF/KCNA3/GABPA/HMGB1/CXCL12/CCL2/CRYAB/IFNB1/APOE/CRP/HLA-C/IL6R/IRF8/NOD2/GAPDH/EIF2B5/ICAM1/IRF5/IRF1/PTPRC/CD6/SLC11A1/ITGA4/PTPRCAP/FCGR3A/HLA-DRB1/CXCR3/GZMB/HLA-DRB5/SELPLG/CD4/GRN/HLA-DRA/CD14
DO	DOID:2377	multiple sclerosis	9.35E-04	AR/GAB1/F8/FGF2/LIG4/HFE/WWOX/APOC1/CYP3A4/AMBP/TET2/BRAF/EIF4E/TNPO1/NSD2/COL1A1/BDNF/CYP3A5/FN1/RAD23B/SPP1/DNMT1/CCNK/GSK3B/PIK3CA/BCL9/RARB/SFRP2/CXCL12/CYP2C19/KRAS/PTN/ETV6/THPO/IGFBP1/PTPN11/LDHC/FRZB/F7/CTAG2/INHBA/SFRP1/BMP2/TNFRSF11B/HGFAC/MBL2/SFRP4/NOTCH1/RHOA/IFNAR1/RASSF1/IL6R/IRF8/CDKN1B/CEBPB/ICAM1/MVP/PTPRC/HCK/TNFSF10/LRP1/CYP1B1/ITGA4/CCL4/XBP1/CD74/NFKBIA/GSTP1/PLAUR/SULF2/SOCS3/CXCR4
DO	DOID:4960	bone marrow cancer	9.73E-04	AR/GAB1/F8/FGF2/LIG4/HFE/WWOX/APOC1/CYP3A4/AMBP/TET2/BRAF/EIF4E/TNPO1/NSD2/COL1A1/BDNF/CYP3A5/FN1/RAD23B/SPP1/DNMT1/CCNK/GSK3B/PIK3CA/BCL9/RARB/SFRP2/CXCL12/CYP2C19/KRAS/PTN/ETV6/THPO/IGFBP1/LDHC/FRZB/F7/CTAG2/INHBA/SFRP1/BMP2/TNFRSF11B/HGFAC/MBL2/SFRP4/NOTCH1/RHOA/IFNAR1/RASSF1/IL6R/IRF8/CDKN1B/CEBPB/ICAM1/MVP/PTPRC/HCK/TNFSF10/LRP1/CYP1B1/ITGA4/CCL4/XBP1/CD74/NFKBIA/GSTP1/PLAUR/SULF2/SOCS3/CXCR4
DO	DOID:0070004	myeloma	1.01E-03	GAB1/FGF2/LIG4/WWOX/APOC1/CYP3A4/AMBP/BRAF/EIF4E/TNPO1/NSD2/COL1A1/BDNF/CYP3A5/RAD23B/SPP1/DNMT1/CCNK/GSK3B/PIK3CA/BCL9/SFRP2/CXCL12/CYP2C19/KRAS/PTN/IGFBP1/FRZB/CTAG2/INHBA/SFRP1/BMP2/TNFRSF11B/HGFAC/MBL2/SFRP4/NOTCH1/RHOA/IFNAR1/RASSF1/IL6R/IRF8/CDKN1B/CEBPB/ICAM1/MVP/PTPRC/HCK/TNFSF10/LRP1/CYP1B1/ITGA4/CCL4/XBP1/CD74/NFKBIA/GSTP1/PLAUR/SULF2/CXCR4
DO	DOID:9538	multiple myeloma	1.01E-03	ITGA1/ATXN2/HFE/C3/C7/MBP/EBF1/APOA1/BDNF/KCNA3/GABPA/HMGB1/CXCL12/CCL2/CRYAB/IFNB1/APOE/CRP/HLA-C/IL6R/IRF8/NOD2/GAPDH/EIF2B5/ICAM1/IRF5/IRF1/PTPRC/CD6/SLC11A1/ITGA4/PTPRCAP/FCGR3A/HLA-DRB1/CXCR3/GZMB/HLA-DRB5/SELPLG/CD4/GRN/HLA-DRA/CD14/ITGB2
DO	DOID:3213	demyelinating disease	1.04E-03	TTC37/SLC26A3/FGF19/APOE/HTR3E/UGT1A1/CFTR/EGFR/SLC9A3/TNFRSF11B/TLR4/S100A8/CD4
DO	DOID:13250	diarrhea	1.07E-03	PAWR/ASPH/CDKN2B/APOB/TTR/WWOX/BRAF/TP53BP1/DNMT3B/SPP1/DNMT1/RPS6KB1/PIK3CA/CXCL12/KRAS/SMAD4/APOE/PROM1/MUC4/WISP1/TFF2/EGFR/NGF/SCTR/RASSF1/IMP3/ANXA2/VIM/TNFSF10/ANXA1/LGALS3/TYMP/SERPINA1/SOCS3/CXCR4/DUSP1
DO	DOID:4606	bile duct cancer	1.74E-04	ALB/FGA/CYP2C8/FGF2/HFE/APOB/FGG/C3/APOC3/APOA1/ADH1B/BDNF/APOH/FGB/FN1/F2R/TUBB1/LRRFIP1/CYP2C9/PON1/PLG/CXCL12/SERPINC1/FABP3/ADH1C/CYP2C19/CCL2/PAPPA/IGFBP1/MEF2A/APOE/PROM1/AHSG/F7/PTGIS/CCL11/ABCC9/UGT1A1/CYP1A2/ADIPOQ/INHBA/F11/CRP/TNFRSF11B/MB/MBL2/TNNT2/ELN/PPIA/TLR4/ALOX5/CTSD/ICAM1/PTPRC/ALOX5AP/ADAM8/GPX1/TLR2/PRF1/CST3/GZMB/PLAUR/CD14/CXCR4/TNFRSF1B/S100A9

*KEGG* Kyoto Encyclopedia of Genes and Genomes, *DO* Disease Ontology

Among these items, the following were related to AMI: GO:0002283 neutrophil activation involved in immune response; GO:0043312 neutrophil degranulation; GO:0042119 neutrophil activation; GO:0002446 neutrophil-mediated immunity; GO:0043405 regulation of MAP kinase activity; DOID:5844 myocardial infarction; DOID:3393 coronary artery disease; DOID:4248 coronary stenosis; hsa04932 non-alcoholic fatty liver disease (NAFLD); hsa05010 Alzheimer’s disease; and hsa04514 cell adhesion molecules (CAMs). The genes included in these terms were selected for further analysis.

**Fig. 5:**
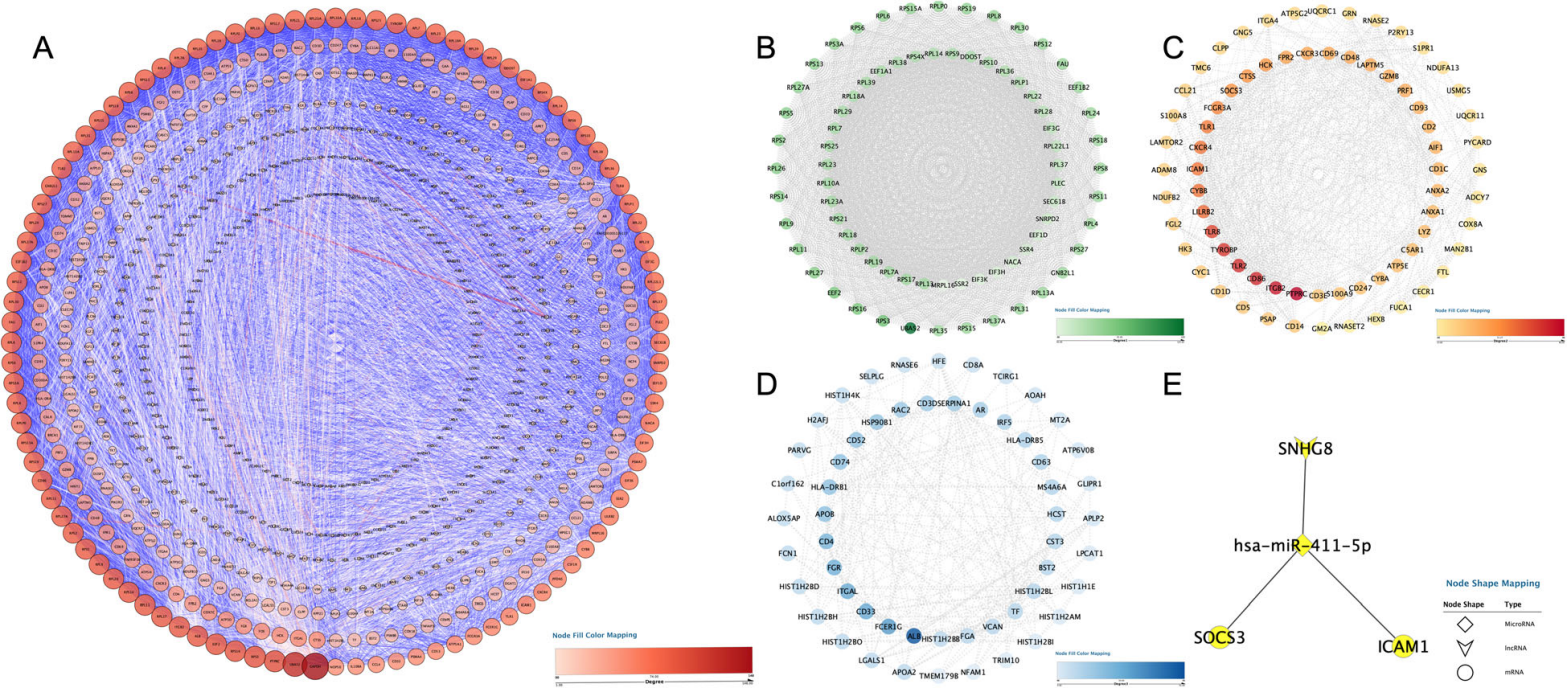
PPI network construction and identification of hub genes**. a** PPI network of the selected DEGs. The edge shows the interaction between two genes. Significant modules identified from the PPI network using the molecular complex detection method with a score > 10. **b** Molecular-1 with MCODE = 87.63; (**c**) Molecular-2 with MCODE = 24.79; (**d**) Molecular-3 with MCODE = 11.86. A degree was used to describe the importance of protein nodes in the network, with a dark color filling denoting a high degree and light color a low degree

To generate a PPI for these DEGs, data analysis was performed using the STRING database, from which 7544 protein pairs and 591 nodes were revealed with a combined score > 0.9. Figure 5a shows the net analysis in Cytoscape. Three modules with a score > 10 were found and are presented in Fig. 5 (B-D) for detection using the Molecular Complex Detection (MCODE) application. These three modules included a total of 300 genes. Finally, after a comprehensive analysis of the GO, DO, and KEGG data, we selected 2 DEGs related to the onset of AMI, which demonstrated a high degree of association simultaneously, as well as in the submodule analysis. These two genes were suppressor of cytokine signaling 3 (SOCS3) and intercellular adhesion molecule 1 (ICAM1) and were located in module 2 (Fig. 5c).

### Construction of the lncRNA-miRNA-mRNA regulatory network

First, considering the interaction between mRNAs (SOCS3 and ICAM1) and miRNAs, the miRwalk databases and DIANA TOOLS were searched for miRNA-mRNA interactions. Subsequently, we predicted lncRNAs (DELs) that can bind with miRNAs to design the lncRNA-miRNA regulatory network using the starBase (v3.0) and miRcode databases. Then, we matched the predicted miRNAs to build a network and found that lncRNA SNHG8, hsa-miR-411-5p, SOCS3 and ICAM1 were hub items in this triple regulatory network. Ultimately, a lncRNA-miRNA-mRNA network was formed by merging the two sets of data and was visualized by Cytoscape (version 3.71) (Fig. 5e).

### The validation of expression profiles

After a comprehensive analysis, lncRNA SNHG8, SOCS3 and ICAM1 were validated in our samples, which included 115 controls and 115 patients who suffered from.

**Fig. 6:**
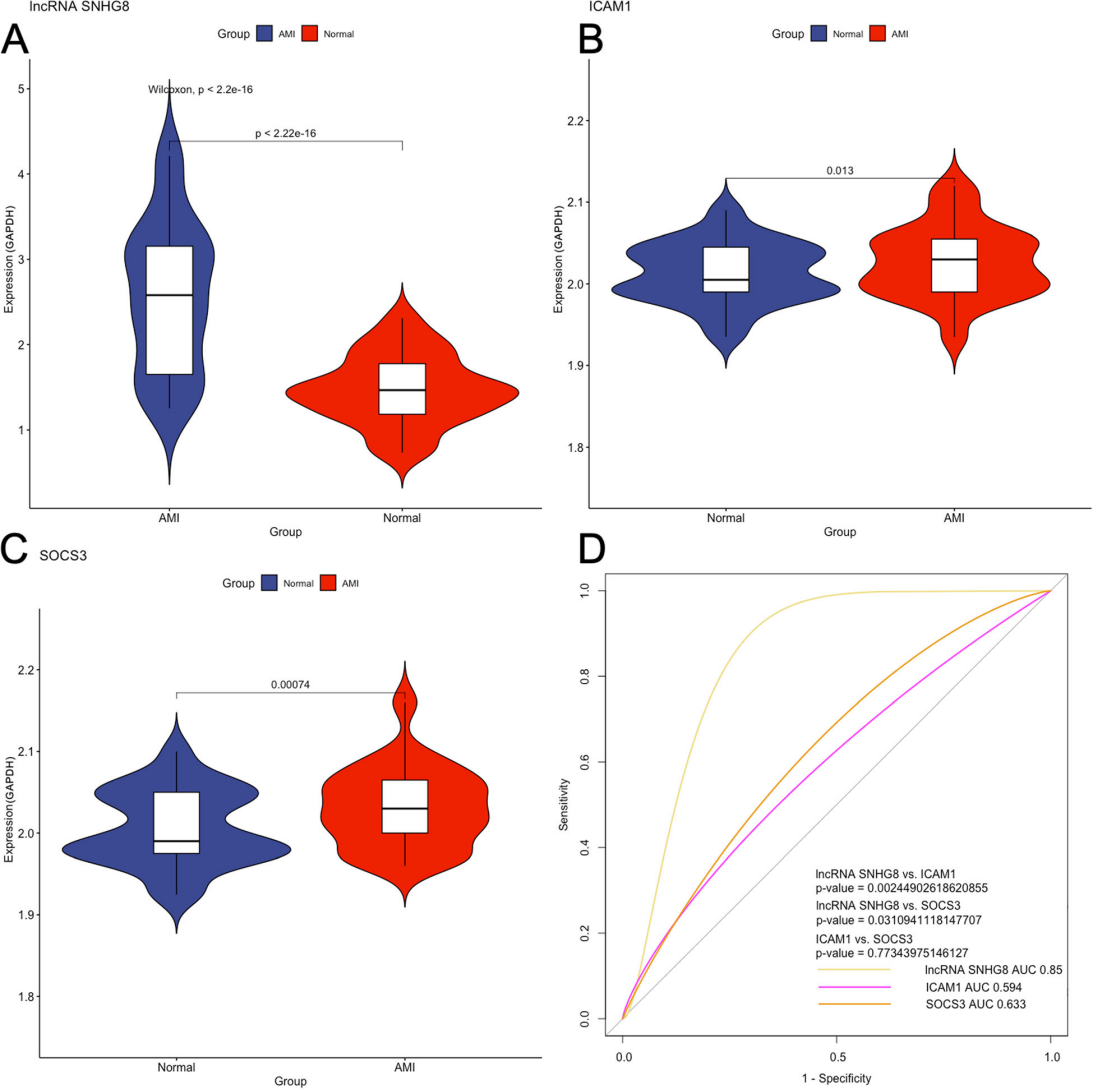
Confirmed expression levels and ROC curve analyses for the diagnosis of AMI. **a**-**c** Relative expression of lncRNA SNHG8, ICAM1 and SOCS3; (**d**) ROC curve analysis and pairwise *P*-value comparison

AMI. The demographic results are shown in Table 3. After validation, we found that the relative expression of lncRNA SNHG8, SOCS3 and ICAM1 was increased in AMI compared with the normal controls with significant differences (Fig. 6a-c). This conclusion was the same as the trend we obtained when we analyzed the RNA-seq data.

**Table 3:** Comparison of demographic and lifestyle characteristics and serum lipid levels between the normal and AMI groups

Parameter	Control	AMI	*test-statistic*	*P*
Number	115	115		
Male/female	35/80	36/79	0.020	0.886
Age (years)^a^	54.3 ± 9.4	55.1 ± 10.1	0.854	0.491
Height (cm)	154.2 ± 6.9	155.5 ± 7.1	1.592	0.212
Weight (kg)	52.9 ± 7.6	60.5 ± 10.8	20.439	1.67E-005
Body mass index (kg/m^2^)	22.76 ± 2.94	23.69 ± 3.28	−2.317	0.021
Waist circumference (cm)	73.4 ± 6.6	87.5 ± 9.9	23.122	3.34E-005
Smoking status [*n* (%)]	29(25.1)	43(37.8)	7.690	0.005
Alcohol consumption [*n* (%)]	27(23.4)	25(22.3)	0.309	0.578
Systolic blood pressure (mmHg)	127.2 ± 18.2	132.5 ± 21.2	0.693	0.231
Diastolic blood pressure (mmHg)	80.5 ± 10.3	81.5 ± 13.2	0.707	0.281
Pulse pressure (mmHg)	47.6 ± 13.1	49.3 ± 13.6	1.582	0.213
Glucose (mmol/L)	5.92 ± 1.81	7.61 ± 2.71	15.867	2.71E-005
Total cholesterol (mmol/L)	4.91 ± 1.23	5.64 ± 1.26	9.122	0.006
Triglyceride (mmol/L)^b^	1.47(0.50)	1.52(1.21)	2.116	0.154
HDL-C (mmol/L)	1.43 ± 0.41	1.16 ± 0.14	8.677	0.014
LDL-C (mmol/L)	2.93 ± 0.82	3.89 ± 0.74	10.491	0.002
ApoA1 (g/L)	1.21 ± 0.25	1.27 ± 0.27	0.381	0.518
ApoB (g/L)	0.86 ± 0.17	0.91 ± 0.22	1.563	0.192
ApoA1/ApoB	1.7 ± 0.5	1.7 ± 0.6	0.095	0.758
Heart rate (beats/minutes)	73.5 ± 10.6	72.2 ± 13.4	0.413	0.44
Creatinine, (μmol/L)	72.61 ± 12.35	75.87 ± 13.88	8.694	0.010
Uric acid, (μmol/L)	282.10 ± 74.64	282.13 ± 72.61	0.273	0.744
Troponin T, (μg/L)	0.01 ± 0.02	1.71 ± 2.81	12.437	1.58E-005
CK, (U/L)	86.30 ± 47.68	1007.07 ± 1502.9	16.812	3.89E-005
CKMB, (U/L)	13.73 ± 4.53	115.85 ± 169.70	17.889	4.76E-005

*HDL-C* high-density lipoprotein cholesterol, *LDL-C* low-density lipoprotein cholesterol, *Apo* Apolipoprotein, *CK* creatine kinase, *CKMB* creatine kinase-myocardial band ^a^Mean ± SD determined by *t*-test. ^b^Because it was not normally distributed, the value of triglyceride content was presented as median (interquartile range), and the difference between the two groups was determined by the Wilcoxon-Mann-Whitney test

### Expression level biomarker sensitivity for AMI

Considering the above mentioned observations, we further assessed these two genes (SOCS3 and ICAM1) and lncRNA SNHG8 as potential biomarkers for AMI. ROC curve analysis showed that lncRNA SNHG8 presented an AUC of 0.85, while the AUC of ICAM1 was 0.594 and that of SOCS3 was 0.633. After the pairwise comparison, we found that lncRNA SNHG8 had significant statistical significance (*P*_SNHG8-ICAM1_ = 0.002; *P*_SNHG8-SOCS3_ = 0.031, Fig. 6d).

### Nomogram prediction model development to estimate individual AMI probability

**Fig. 7:**
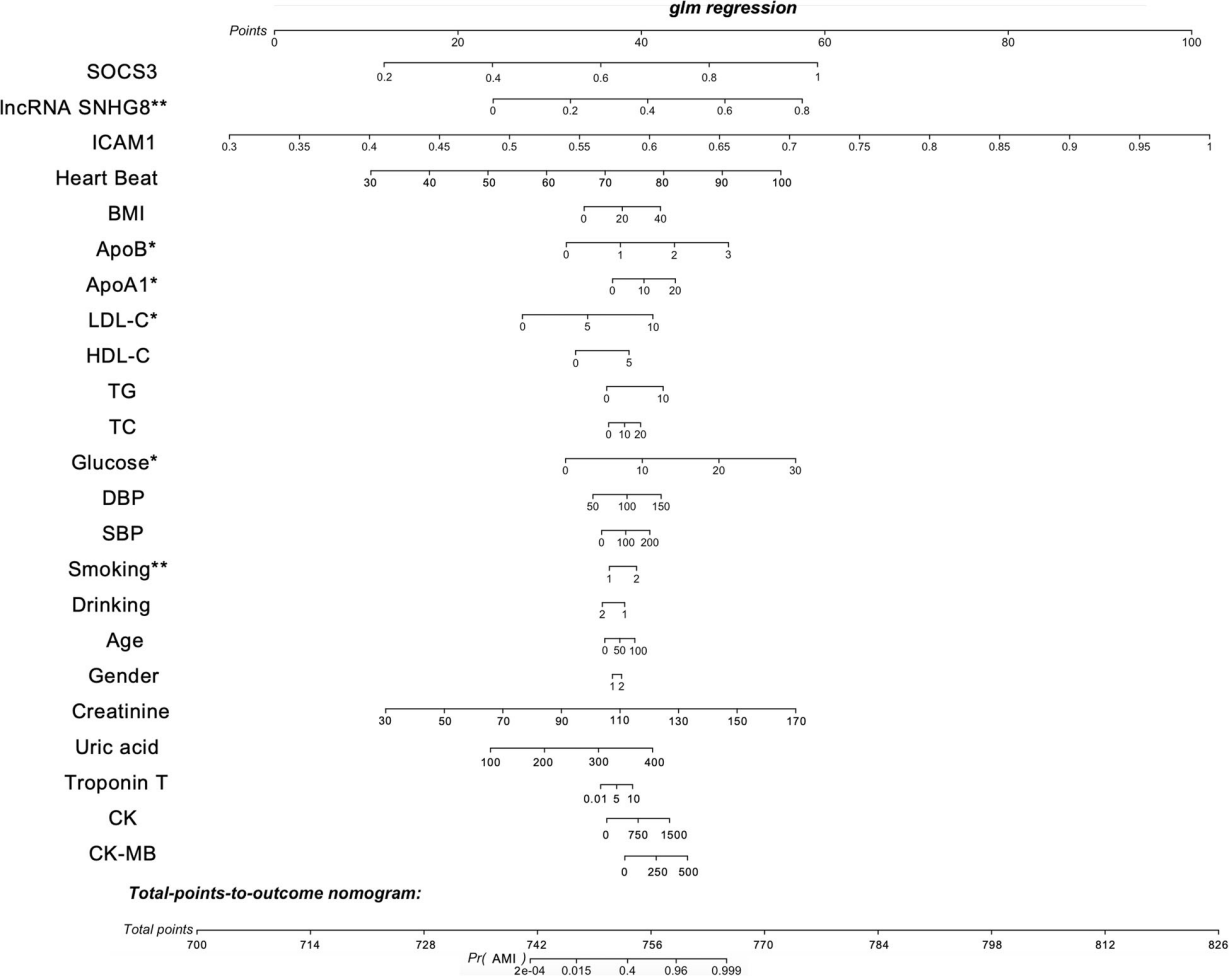
Nomogram to estimate individual AMI probability. The nomogram was constructed based on a logistic regression model for the outcome of definite AMI. Each predictor variable characteristic had a corresponding point value based on its position on the top point scale and contribution to the model. The probability of AMI for each subject was calculated by adding the points for each variable to obtain a total point value that corresponded to a probability of AMI from the scale presented on the bottom line. *P < 0.05, **P < 0.01

We selected gender, age, smoking, drinking, BMI, SBP, DBP, serum glucose, TC, TG, HDL-C, LDL-C, ApoA1, ApoB, heartbeat, creatinine, uric acid, troponin T, CK, and CKMB, and the relative expression of lncRNA SNHG8, SOCS3, and ICAM1 were the best subset of risk factors to develop an AMI risk score and risk model (nomogram) (Fig. 7). In this analysis, male was labeled as 1 and female was labeled as 2; and for smoking and drinking, yes was labeled as 2 and no was labeled 1. The nomogram had excellent discriminative power based on the C-statistic and was well calibrated with the Hosmer-Lemeshow χ 2 statistic. The predicted probabilities of developing AMI ranged from 0.00002 to 99.9%. After calculation, lncRNA SNHG8, ApoB, ApoA1, LDL-C, serum glucose and smoking were statistically significantly related to the risk of AMI.

## Discussion

Cardiovascular disease is currently the leading cause of death. The number of cardiovascular patients, especially those with acute myocardial infarction, will continue to grow steadily in the next 10 years [30]. Traditional prevention of myocardial infarction includes anti-platelet and lipid regulation. Unfortunately, statins are frequently not available for several reasons. Nutraceuticals and functional food ingredients that are beneficial to vascular health may represent useful compounds that are able to reduce the overall cardiovascular risk induced by dyslipidemia by acting parallel to statins or as adjuvants in case of failure or in situations where statins cannot be used [31]. In-depth study of the occurrence and development mechanism of AMI and a search for reliable predictive biomarkers will have a great impact on the treatment and prevention of AMI. In recent years, with the continuous progress of technology, high-throughput sequencing technology has been widely used to analyze the causes of diseases and to find reliable predictive biomarkers.

Recently, an increasing number of studies have demonstrated that lncRNAs participate in fundamental cellular processes, such as gene transcription, post transcriptional gene regulation, RNA processing, gene regulation and chromatin modification [32]. In addition, lncRNAs can also play a role as competing endogenous RNAs (ceRNAs) by sponging specific miRNAs to release their target mRNAs [33]. Moreover, many lncRNAs have been found to be modulators in the progression of cardiovascular diseases (CVDs), such as cardiovascular aging, myocardial infarction and cardiac hypertrophy [34, 35]. Moreover, several previous studies have reported that lncRNAs can regulate biological processes that are associated with myocardial infarction [36]. In our current study, we found that the relative expression of lncRNA SNHG8 was upregulated in AMI. SNHG8, located on 4q26, is thought to encode small nucleolar RNAs (snoRNAs) that function as targets of transcription factor FoxM1 in the regulation of muscle satellite cell proliferation and survival [37]. SNHG8 may be related to the regulation of myocardial muscle cell necrosis after acute myocardial ischemia.

MicroRNAs (miRNAs), which are conserved 19–25 nucleotide noncoding RNAs that function in regulating post-translational gene expression, have become a focus of translational research [38]. Recently, several studies have found that miR-411 is associated with metabolic diseases and atherosclerosis-related diseases. Zhao et al. found that hsa-miR-411-5p was associated with high-fat diet-induced hepatic insulin resistance in mice [39]. Stather et al. demonstrated that hsa-miR-411 was related to peripheral arterial disease and atherosclerosis [40]. According to database prediction, miR-411-5p may be a target that binds to SOCS3 and ICAM1. These two genes have been confirmed to be associated with myocardial infarction [41, 42].

Recently, lncRNA–miRNA–mRNA axes have been shown to be unique regulatory mechanisms that are closely related to cardiovascular diseases (CVDs). For instance, nuclear factor IA (NFIA) regulates cholesterol homeostasis in the body and promotes the progression of atherosclerosis through the lncRNA RP5833A20.1 sponging miR-382-5p, which targets NFIA axis [43]. These findings suggest that lncRNAs could become candidate clinical diagnostic and prognostic markers, providing new therapeutic targets for CVDs and future insights into the prevention and treatment of other diseases. In our current study, we also found that the relative expression of lncRNA SNHG8 was significantly elevated in AMI and could be used as a promising biomarker for the diagnosis and treatment for AMI. The specific mechanism may be that lncRNA SNHG8 could regulate SOCS3 or ICAM1 expression by sponging hsa-miR-411-5p in AMI.

This study has its limitations. First, the patients enrolled in this study to validate the relative expression were from only one hospital, and the sample size may be a little small. Whether there are differences for patients from different areas and races is not known. Therefore, the validity of the results should be tested further in additional prospective cohorts. Second, the specific mechanism of the lncRNA-miRNA-mRNA axes for regulating the pathogenesis of CAD has not been validated in vivo and in vitro.

## Conclusion

In conclusion, we explored the molecular mechanism of AMI by constructing the lncRNA-miRNA-mRNA axis based on the ceRNA hypothesis. After analyzing RNA-seq data, we combined differentially expressed mRNAs and lncRNAs. After functional analysis and predictive ncRNA network construction, we identified the lncRNA SNHG8-miR-411-5p-SOCS3/ICAM1 regulatory network. LncRNA SNHG8 may regulate SOCS3 or ICAM1 expression by sponging hsa-miR-411-5p in AMI and may serve as diagnostic or prognostic biomarkers of AMI.

## Additional file

**Additional file 1: Table S1.** The primers used in qPCR of the lncRNA and mRNAs.

## Data Availability

The datasets used and/or analysed during the current study are available from the corresponding author on reasonable request.

## Abbreviations

AMI: Acute myocardial infarction
Apo: Apolipoprotein
BMI: Body mass index
DBP: Diastolic blood pressure
DEGs: Differentially expressed mRNAs
DELs: Differentially expressed lncRNAs
DEMis: Differentially expressed miRNAs
DO: Disease ontology
HDL-C: High-density lipoprotein cholesterol
LDL-C: Low-density lipoprotein cholesterol
MCODE: Molecular complex detection
PP: Pulse pressure
RMA: Robust Multi-array Average
ROC: Receiver operating characteristic
SBP: Systolic blood pressure
TC: Total cholesterol
TG: Triglyceride
WC: Waist circumference

## References

[1] Reindl M, Reinstadler SJ, Feistritzer HJ, Mayr A, Klug G, Marschang P, Metzler B (2016). Acute myocardial infarction as a manifestation of systemic vasculitis. Wien Klin Wochenschr.

[2] Cervellin G, Rastelli G (2016). The clinics of acute coronary syndrome. Ann Transl Med.

[3] Chiu MH, Heydari B, Batulan Z, Maarouf N, Subramanya V, Schenck-Gustafsson K, O'Brien ER (2018). Coronary artery disease in post-menopausal women: are there appropriate means of assessment?. Clin Sci (Lond).

[4] Madhavan MV, Gersh BJ, Alexander KP, Granger CB, Stone GW (2018). Coronary artery disease in patients >/=80 years of age. J Am Coll Cardiol.

[5] Abram S, Arruda-Olson AM, Scott CG, Pellikka PA, Nkomo VT, Oh JK, Milan A, Abidian MM, McCully RB. Frequency, predictors, and implications of abnormal blood pressure responses during Dobutamine stress echocardiography. Circ Cardiovasc Imaging. 2017;10. 10.1161/CIRCIMAGING.116.005444 PMC5408460 28351907

[6] Yamada Y, Matsui K, Takeuchi I, Fujimaki T (2015). Association of genetic variants with coronary artery disease and ischemic stroke in a longitudinal population-based genetic epidemiological study. Biomed Rep.

[7] Aryal B, Rotllan N, Fernandez-Hernando C (2014). Noncoding RNAs and atherosclerosis. Curr Atheroscler Rep.

[8] Fu XD (2014). Non-coding RNA: a new frontier in regulatory biology. Natl Sci Rev.

[9] Elia L, Condorelli G (2015). RNA (epi)genetics in cardiovascular diseases. J Mol Cell Cardiol.

[10] Dechamethakun S, Muramatsu M (2017). Long noncoding RNA variations in cardiometabolic diseases. J Hum Genet.

[11] Quinn JJ, Chang HY (2016). Unique features of long non-coding RNA biogenesis and function. Nat Rev Genet.

[12] Zhong Z, Hou J, Zhang Q, Li B, Li C, Liu Z, Yang M, Zhong W, Zhao P (2018). Differential expression of circulating long non-coding RNAs in patients with acute myocardial infarction. Medicine (Baltimore).

[13] Song YX, Sun JX, Zhao JH, Yang YC, Shi JX, Wu ZH, Chen XW, Gao P, Miao ZF, Wang ZN (2017). Non-coding RNAs participate in the regulatory network of CLDN4 via ceRNA mediated miRNA evasion. Nat Commun.

[14] Eicher JD, Wakabayashi Y, Vitseva O, Esa N, Yang Y, Zhu J, Freedman JE, McManus DD, Johnson AD (2016). Characterization of the platelet transcriptome by RNA sequencing in patients with acute myocardial infarction. Platelets.

[15] Gentleman RC, Carey VJ, Bates DM, Bolstad B, Dettling M, Dudoit S, Ellis B, Gautier L, Ge Y, Gentry J (2004). Bioconductor: open software development for computational biology and bioinformatics. Genome Biol.

[16] Miao L, Yin RX, Zhang QH, Hu XJ, Huang F, Chen WX, Cao XL, Wu JZ. Integrated DNA methylation and gene expression analysis in the pathogenesis of coronary artery disease. Aging (Albany NY). 2019. 10.18632/aging.101847 PMC6428103 30844764

[17] Yu G, Wang LG, Han Y, He QY (2012). clusterProfiler: an R package for comparing biological themes among gene clusters. OMICS.

[18] Szklarczyk D, Gable AL, Lyon D, Junge A, Wyder S, Huerta-Cepas J, Simonovic M, Doncheva NT, Morris JH, Bork P (2019). STRING v11: protein-protein association networks with increased coverage, supporting functional discovery in genome-wide experimental datasets. Nucleic Acids Res.

[19] Miao L, Yin RX, Pan SL, Yang S, Yang DZ, Lin WX (2019). Circulating miR-3659 may be a potential biomarker of dyslipidemia in patients with obesity. J Transl Med.

[20] Shannon P, Markiel A, Ozier O, Baliga NS, Wang JT, Ramage D, Amin N, Schwikowski B, Ideker T (2003). Cytoscape: a software environment for integrated models of biomolecular interaction networks. Genome Res.

[21] Bader GD, Hogue CW (2003). An automated method for finding molecular complexes in large protein interaction networks. BMC Bioinformatics.

[22] Dweep H, Gretz N (2014). Sticht C: miRWalk database for miRNA-target interactions. Methods Mol Biol.

[23] Paraskevopoulou MD, Georgakilas G, Kostoulas N, Vlachos IS, Vergoulis T, Reczko M, Filippidis C, Dalamagas T, Hatzigeorgiou AG (2013). DIANA-microT web server v5.0: service integration into miRNA functional analysis workflows. Nucleic Acids Res.

[24] Jeggari A, Marks DS (2012). Larsson E: miRcode: a map of putative microRNA target sites in the long non-coding transcriptome. Bioinformatics.

[25] Li JH, Liu S, Zhou H, Qu LH, Yang JH (2014). starBase v2.0: decoding miRNA-ceRNA, miRNA-ncRNA and protein-RNA interaction networks from large-scale CLIP-Seq data. Nucleic Acids Res.

[26] Thygesen K, Alpert JS, Jaffe AS, Chaitman BR, Bax JJ, Morrow DA, White HD (2018). Executive group on behalf of the joint European Society of Cardiology /American College of Cardiology /American Heart Association /world heart federation task force for the universal definition of myocardial I: fourth universal definition of myocardial infarction (2018). Circulation.

[27] Miao L, Yin RX, Yang S, Huang F, Chen WX, Cao XL (2017). Association between single nucleotide polymorphism rs9534275 and the risk of coronary artery disease and ischemic stroke. Lipids Health Dis.

[28] Miao L, Yin RX, Huang F, Chen WX, Cao XL, Wu JZ (2017). The effect of MVK-MMAB variants, their haplotypes and GxE interactions on serum lipid levels and the risk of coronary heart disease and ischemic stroke. Oncotarget.

[29] Miao L, Yin RX, Pan SL, Yang S, Yang DZ, Lin WX (2018). Weighted gene co-expression network analysis identifies specific modules and hub genes related to hyperlipidemia. Cell Physiol Biochem.

[30] Chen ZH, Zhang M, Li YC, Zhao ZP, Zhang X, Huang ZJ, Li C, Wang LM (2018). Study on relationship between prevalence or co-prevalence of risk factors for cardiovascular disease and blood pressure level in adults in China. Zhonghua Liu Xing Bing Xue Za Zhi.

[31] Scicchitano P, Cameli M, Maiello M (2014). Nutraceuticals and dyslipidaemia: beyond the common therapeutics. J Funct Foods.

[32] Orom UA, Derrien T, Beringer M, Gumireddy K, Gardini A, Bussotti G, Lai F, Zytnicki M, Notredame C, Huang Q (2010). Long noncoding RNAs with enhancer-like function in human cells. Cell.

[33] Wang M, Mao C, Ouyang L, Liu Y, Lai W, Liu N, Shi Y, Chen L, Xiao D, Yu F, et al. Long noncoding RNA LINC00336 inhibits ferroptosis in lung cancer by functioning as a competing endogenous RNA. Cell Death Differ. 2019. 10.1038/s41418-019-0304-y PMC6889193 30787392

[34] Ounzain S, Micheletti R, Beckmann T, Schroen B, Alexanian M, Pezzuto I, Crippa S, Nemir M, Sarre A, Johnson R (2015). Genome-wide profiling of the cardiac transcriptome after myocardial infarction identifies novel heart-specific long non-coding RNAs. Eur Heart J.

[35] Wang K, Liu F, Zhou LY, Long B, Yuan SM, Wang Y, Liu CY, Sun T, Zhang XJ, Li PF (2014). The long noncoding RNA CHRF regulates cardiac hypertrophy by targeting miR-489. Circ Res.

[36] Liu CY, Zhang YH, Li RB, Zhou LY, An T, Zhang RC, Zhai M, Huang Y, Yan KW, Dong YH (2018). LncRNA CAIF inhibits autophagy and attenuates myocardial infarction by blocking p53-mediated myocardin transcription. Nat Commun.

[37] Chen Z, Bu N, Qiao X, Zuo Z, Shu Y, Liu Z, Qian Z, Chen J, Hou Y (2018). Forkhead box M1 transcriptionally regulates the expression of Long noncoding RNAs Snhg8 and Gm26917 to promote proliferation and survival of muscle satellite cells. Stem Cells.

[38] Feinberg MW, Moore KJ (2016). MicroRNA regulation of atherosclerosis. Circ Res.

[39] Zhao X, Chen Z, Zhou Z, Li Y, Wang Y, Zhou Z, Lu H, Sun C, Chu X (2019). High-throughput sequencing of small RNAs and analysis of differentially expressed microRNAs associated with high-fat diet-induced hepatic insulin resistance in mice. Genes Nutr.

[40] Stather PW, Sylvius N, Sidloff DA, Dattani N, Verissimo A, Wild JB, Butt HZ, Choke E, Sayers RD, Bown MJ (2015). Identification of microRNAs associated with abdominal aortic aneurysms and peripheral arterial disease. Br J Surg.

[41] Ge WH, Lin Y, Li S, Zong X, Ge ZC (2018). Identification of biomarkers for early diagnosis of acute myocardial infarction. J Cell Biochem.

[42] Zhang Y, Shao T, Yao L, Yue H, Zhang Z (2018). Effects of tirofiban on stent thrombosis, Hs-CRP, IL-6 and sICAM-1 after PCI of acute myocardial infarction. Exp Ther Med.

[43] Hu YW, Zhao JY, Li SF, Huang JL, Qiu YR, Ma X, Wu SG, Chen ZP, Hu YR, Yang JY (2015). RP5-833A20.1/miR-382-5p/NFIA-dependent signal transduction pathway contributes to the regulation of cholesterol homeostasis and inflammatory reaction. Arterioscler Thromb Vasc Biol.

