# Supplementary material for: LncRNA SNHG8 is identified as a key regulator of acute myocardial infarction by RNA-seq analysis
Source: Lipids Health Dis. 2019 Nov 18;18:201. doi: 10.1186/s12944-019-1142-0 (PMC6862811; doi:10.1186/s12944-019-1142-0)
Supplement: Supplementary file 1 — Additional file 1: Table S1. The primers used in qPCR of the lncRNA and mRNAs. [file 12944_2019_1142_MOESM1_ESM.docx]

**Additional file 1: Table S1.** The primers used in qPCR of the lncRNA and mRNAs.

| LncRNA/mRNA | Forward primer | Reverse primer |
| --- | --- | --- |
| SNHG8 | CCCGAGAACCGTCAGTTTGA | CCGGCACCCTCTAGGTTTTT |
| ICAM1 | GCCCGATTGCTTTAGCTTGG | CAATCGCTGTCTCTAGCCCC |
| SOCS3 | TCGGAGGAGGGTTCAGTAGG | AGGGTTCCGGGCACTCAA |
| GAPDH | AGGTCGGTGTGAACGGATTTG | GGGGTCGTTGATGGCAACA |
